# CanScreen5, a global repository for breast, cervical and colorectal cancer screening programs

**DOI:** 10.1038/s41591-023-02315-6

**Published:** 2023-04-27

**Authors:** Li Zhang, Isabel Mosquera, Eric Lucas, Mary Luz Rol, Andre L. Carvalho, Partha Basu, CanScreen5 collaborators, Daniel Sadowski, Bartlett Natasha, Alison Budd, Ashrafun Nessa, Isabel De Brabander, Annemie Haelens, Sarah Pringels, Jonas Tairo, Solveig Hofvind, J. B. Burrion, Zdravka Valerianova, Jill Tinmouth, Cindy Law, Simbi Ebenuwah, Bronwen McCurdy, Beata Janik, George Pupwe, Linn Fenna Groeneveld, Gry Baadstrand Skare, Penelope Layne, Tytti Sarkeala, Mwate Joseph Chaila, Michal Kaminski, Beata Kinel, Jolanta Lissowska, Inga Mumukunde, Vitor Rodrigues, Robinson Rodríguez, Elena Pérez Sanz, Raquel Zubizarreta Alberdi, Guglielmo Ronco, Nataša Antoljak, Dinka Nakić, Davor Plazanin, Andrea Šupe Parun, Mat Goossens, Andrzej Nowakowski, Harry de Koning, Els Dams, Asha Martin, Mara Epermane, Nataļja Jankovska, Scott Antle, Jacques Fracheboud, Esther Toes-Zoutendijk, Heleen M. E. van Agt, Karen Budewig, Barbara Stomper, Ahti Anttila, Sorana McLeish, Alexandra Ramssl-Sauer, Georg Ziniel, Theopisti Kyprianou, Pavlos Pavlou, Fofo Kaliva, Maria Tsantidou, Shaokai Zhang, Huifang Xu, Wali Mushtaq, Biviana Paredes Barragán, Alexandra Montalvo, Ana Victoria de la Torre Santos, Marie-Helene Guertin, Sarah Fournier, Nicolas Duport, Ondřej Májek, Ondřej Ngo, Urška Ivanuš, Katja Jarm, Maja Primic-Zakelj, Flávia de Miranda Corrêa, Arn Migowski, Marianna Cancela, Patricia Gallardo, Gisel Fattore, Adrián Puello, Víctor Polanco, Nieves Ascunce Elizaga, Valerie Fabri, Paola Mantellini, Marco Zappa, Eliane Kellen, Elsebeth Lynge, Vanessa Kääb-Sanyal, Daniela Malek, Youssef Chami, Rugile Ivanauskiene, Nensy Bandhoe, Claire Dillenbourg, Karin Heckters, Astrid Scharpantgen, Oris Mariela Ruiz, Geneva Mireya González, Elsa Arenas, Eduardo Alberto Palacios Cacacho, Alicia Pomata, Eliza Navarro, Milva Mencia, Gisela Abreu Ruiz, Ruth Campoverde, Claudia Camel, Rocío Donis, Yolanda Inés Sandoval, Heidy García, Omaira Isabel Roldán, Teresa del Carmen Moreno, Mario Morales Velado, Gina Merino, Juvenal A. Ríos, Sabrina Marte, Reina Oliva Hernández, Marina Anea Chacón, Xiomara Isabel Ruiz, Xiomara del Carmen Hernández Vivas, Roger Iván González, Damaris Isabel Medal Ruiz, Lourdes Ortega, Andrea Matos Orbegozo, Carlos Adolfo Chuquiyauri Haro, Miriam Dalmas, Fahriye Unlu, Loubna Abousselham, Rose-Marie De Waldt, Charlotte Buys, Yasine Hanna, Cathi-Ann Williams, Londi-Ann Ottey, Shana Philbert-Cyr, Crissah Emmanuel, Alexandra Jemmott, Nuno Augusto Alberto de Miranda, Stala Kioupi, Takelech Moges Asnake, Adel A. Attia, Gontse Tshisimogo, Lame Seema, Jonathan Chiwanda Banda, Feisul Idzwan Mustapha, Rosita Paulo Mugolo, Reginaldo de Alice Miguel Juliao, Mary Nyangasi, Valerian Mwenda, Sonia Tavares Ferreira, Carla Barbosa, Manala Makua, Yacubu Hervé Julius Bakare, Myanna Charles, Leandra Charles, Vera Edwards-France, Cheshta Sewtahal, Bridget Kebirungi, Mugabe Frank Rwabinumi, Valarie Williams, Oritta Zachariah, Arlitha Scott, Camille Deleveaux, Martin Campbell, Cesaltina Ferreira, Suraj Perera, Padmaka Silva, Vindya Kumarapeli, Merisa Grant-Tate, Cherie Tulloch, Kumiko Saika, Kyeongmin Lee, Jae Kwan Jun, Sona Franklin Mukete, Plamen Dimitrov, Vaida Momkuviene, Viačeslavas Zaksas, Piret Veerus, Tatjana Kofol Bric, Dominika Novak Mlakar, Ana Lucija Škrjanec, Jožica Maučec Zakotnik, Tamar Skhirtladze, Stephanie Xuereb, Carol Colquhoun, Suleeporn Sangrajrang, Kumar Eshwar, Greig Stanners, Roger Black, Trude Andreassen, Andras Budai, Lajos Döbrõssy, Attila Kovács, Florian Nicula, Isabel Portillo, Jone Miren Altzibar, Edurne Arenaza, Ndabaningi Simango, Josep A. Espinàs, Constance Glinton-Rolle, Jeanette Anews-Barr, Mariana Capote, Marisa Fazzino, Claire Armstrong, Andrew Gamble, Kenneth Mc Innes, Suzanne Wright, Helen Clayton, Radoslav Latinovic, Anne Mackie, Janet Rimmer, Clare Hall, Klara Miriam Elfström, Sven Törnberg, Manuel Zorzi, Marcis Leja, Dace Rezeberga, Marc Hagenimana, Françoise Hamers, Agnès Rogel, Frank Assogba, Patricia Fitzpatrick, Marc Arbyn, Jacqueline Figueroa, Alex Rovelo, Erosloa Salinas, Elías Yused Argüello, Adriana Milano Castillo, Velia Rosas, Stanislav Špánik, Merete Rønmos Houmann, Dorte Johansen, Joakim Dillner, Kunal Oswal, Yubei Huang, Ismail M. Siala, Michel Candeur, Sarah Hoeck, Josep M. Borras, Luc Bleyen, Bothwell Takaingofa Guzha, Adie Yao Mesmin Olivier, Elisabeth Fasching, Alexander Gollmer Gesundheit Österreich Vienna, Tonoy Taohid, Walkiria Bermejo Bencomo, Darbelis Tejada, Joseph Psaila Valletta, Judy Nisbett, Damaris Baptiste, Melanie Ann Layne

**Affiliations:** 1 International Agency for Research on Cancer, Lyon, France; 2 Alberta Colorectal Cancer Screening Program, Edmonton, Alberta Canada; 3 Australian Institute of Health and Welfare, Canberra, Australian Capital Territory Australia; 4 Bangabandhu Sheikh Mujib Medical University, Dhaka, Bangladesh; 5 Belgian Cancer Registry, Brussels, Belgium; 6 Benjamin Mkapa Hospital, Dar es Salaam, Dodoma, United Republic of Tanzania; 7 Breast Screen Norway, Oslo, Norway; 8 Brumammo, Brussels, Belgium; 9 Bulgarian National Cancer Registry, Sofia, Bulgaria; 10 Canadian Screening for CRC Research Network, Oakville, Ontario Canada; 11 Cancer Care Manitoba, Winnipeg, Manitoba Canada; 12 Cancer Care Ontario, Toronto, Ontario Canada; 13 Cancer Center and Maria Skłodowska-Curie Institute of Oncology, Warsaw, Poland; 14 Cancer Diseases Hospital, Lusaka, Zambia; 15 Cancer Registry Norway, Oslo, Norway; 16 Cancer Registry, Georgetown, Guyana; 17 Cancer Society of Finland, Helsinki, Finland; 18 Catholic Relief Services, Lusaka, Zambia; 19 Clinton Health Acces Initiative, Kigali, Rwanda; 20 Coimbra University/Liga Portuguesa Contrao Cancro, Coimbra, Portugal; 21 Comision Honoraria de Lucha Contra el Cáncer, Montevideo, Uruguay; 22 Conselleria de Sanitat Universali Salut Pública–Generalitat Valenciana, Valencia, Spain; 23 Conselleria de Sanidade Xuntade Galicia Santiago de Compostela, A Coruña, Spain; 24 Centro di Riferimento per l’Epidemiologia e la Prevenzione Oncologica in Piemonte and University Hospital ‘Città della Salute e della Scienza’, Turin, Italy; 25 Croatian Institute of Public Health, Zagreb, Croatia; 26 Centrum voor Kankeropsporing, Bruges, Belgium; 27 Department of Gynaecology and Oncologic Gynaecology, Military Institute of Medicine, Warsaw, Poland; 28 Department of Public Health, Erasmus MC, Rotterdam, the Netherlands; 29 Diakonessen Hospital, Paramaribo, Suriname; 30 Dominica China Friendship Hospital, Roseau, Dominica; 31 East Riga Clinical University Hospital, Riga, Latvia; 32 Eastern Health, Saint John’s, Newfoundland and Labrador Canada; 33 Erasmus Medical Centre, Rotterdam, the Netherlands; 34 Federal Ministry of Health, Bonn, Germany; 35 Finnish Cancer Registry/Cancer Society of Finland, Helsinki, Finland; 36 General Hospital, Saint George’s, Grenada; 37 Gesundheit Österreich, Vienna, Austria; 38 Health Monitoring Unit, Ministry of Health, Nicosia, Cyprus; 39 Hellenic Ministry of Health, Athens, Greece; 40 Henan Cancer Hospital, Zhengzhou, China; 41 Hospital de Especialidades Eugenio Espejo, Quito, Ecuador; 42 Hospital Enrique Garcés, Quito, Ecuador; 43 Hospital General Pablo Arturo Suárez, Quito, Ecuador; 44 Hospital Provincial de Villa Clara, Santa Clara, Cuba; 45 Institut National de Santé Publique du Quebec, Quebec City, Quebec Canada; 46 Institut de Veille Sanitaire Saint-Maurice, Saint-Maurice, France; 47 Institute of Health Information and Statistics of the Czech Republic, Prague, Czech Republic; 48 Institute of Oncology Ljubljana, Ljubljana, Slovenia; 49 Instituto Nacional de Câncer, Rio de Janeiro, Brazil; 50 Instituto Nacional del Cáncer, Buenos Aires, Argentina; 51 Instituto Nacional del Cáncer Rosa Emilia Sánchez Pérez de Tavarez, Santo Domingo, Dominican Republic; 52 Instituto de Salud Públicay Laboral de Navarra Pamplona, Navarra, Spain; 53 Intermutualistic Agency, Brussels, Belgium; 54 Istituto per lo Studio e la Prevenzione Oncologica, Florence, Italy; 55 Katholieke Universiteit Leuven, Leuven, Belgium; 56 Københavns Universitet Copenhagen, Copenhagen, Denmark; 57 Kooperationsgemeinschaft Mammographie, Berlin, Germany; 58 Lalla Salma Foundation-Cancer Prevention and Treatment, Rabat, Morocco; 59 Lithuanian University of Health Sciences Kaunas, Kaunas, Lithuania; 60 Lobi Health Center Foundation, Paramaribo, Suriname; 61 Ministère de la Santé, Luxembourg, Luxembourg; 62 Ministerio de Salud, Panama City, Panama; 63 Ministerio de Salud Pública y Asistencia Social, Guatemala City, Guatemala; 64 Ministerio de Salud Pública y Bienestar Social, Asunción, Paraguay; 65 Ministerio de Salud Pública, La Habana, Cuba; 66 Ministerio de Salud Pública, Quito, Ecuador; 67 Ministerio de Salud y Asistencia Social, Guatemala City, Guatemala; 68 Ministerio de Salud y Protección Social, Bogotá, Colombia; 69 Ministerio de Salud, Unidad Nacional para la Prevención y Control del Cáncer San Salvador, San Salvador, El Salvador; 70 Ministerio de Salud, Metropolitana, Chile; 71 Ministerio de Salud, Santo Domingo, Dominican Republic; 72 Ministerio de Salud, San Salvador, El Salvador; 73 Ministerio de Salud, Managua, Nicaragua; 74 Ministerio de Salud, Lima, Peru; 75 Ministry for Energy and Health, Valletta, Malta; 76 Ministry of Health, Ankara, Turkey; 77 Ministry of Health and Social Protection, Rabat, Morocco; 78 Ministry of Health and Social Services, Windhoek, Namibia; 79 Ministry of Health and Wellness, Kingston, Jamaica; 80 Ministry of Health and Wellness, Castries, Saint Lucia; 81 Ministry of Health, Lisbon, Portugal; 82 Health Monitoring Unit, Ministry of Health Nicosia, Nicosia, Cyprus; 83 Ministry of Health, Addis Ababa, Ethiopia; 84 Ministry of Health, Benghazi, Libya; 85 Ministry of Health, Gaborone, Botswana; 86 Ministry of Health, Lilongwe, Malawi; 87 Ministry of Health, Putrajaya, Malaysia; 88 Ministry of Health, Maputo, Mozambique; 89 Ministry of Health, Nairobi, Kenya; 90 Ministry of Health, Praia, Republic of Cabo Verde; 91 Ministry of Health, Pretoria, South Africa; 92 Ministry of Health, Public Hygiene and Universal Health Coverage, Abidjan, Côte d’Ivoire; 93 Ministry of Health, Saint George’s, Grenada; 94 Health Promotion Unit, Ministry of Health, Basseterre, Saint Kitts and Nevis; 95 Ministry of Health, Paramaribo, Suriname; 96 Ministry of Health, Kampala, Uganda; 97 Ministry of Health, Wellness and the Environment, Saint John’s, Antigua and Barbuda; 98 Ministry of Health, Kingstown, Saint Vincent and the Grenadines; 99 Ministry of Health, Nassau, Bahamas; 100 Ministry of Health, Georgetown, Guyana; 101 Ministry of Health, Maputo, Mozambique; 102 Ministry of Health, Colombo, Sri Lanka; 103 Ministry of Health, Basseterre, Saint Kitts and Nevis; 104 Mount St. John’s Medical Centre Antigua, Saint John’s, Antigua and Barbuda; 105 National Cancer Center, Tokyo, Japan; 106 National Cancer Center, Gyeonggi-do, Republic of Korea; 107 National Cancer Control Committee, Yaounde, Cameroon; 108 National Center of Public Health and Analyses, Sofia, Bulgaria; 109 National Health Insurance Fund, Ministry of Health, Vilnius, Lithuania; 110 National Institute for Health Development, Tallinn, Estonia; 111 National Institute of Public Health, Ljubljana, Slovenia; 112 National Screening Center, Tbilisi, Georgia; 113 National Screening Programmes, Ministry for Energy and Health, Valletta, Malta; 114 National Specialist and Screening Services Directorate Procurement Commissioning and Facilities, Edinburgh, Scotland; 115 National Cancer Institute, Bangkok, Thailand; 116 New Brunswick Cancer Network, Fredericton, New Brunswick Canada; 117 NHS National Services Scotland, Edinburgh, Scotland; 118 Norwegian Directorate of Health, Oslo, Norway; 119 Office of the Chief Medical Officer, Budapest, Hungary; 120 Oncology Institute, Romanian NV Regional Cervical Cancer Screening Programme Management Unit, Cluj-Napoca, Romania; 121 Osakidetza-Servicio Vasco de Salud, Basque Country, Spain; 122 Parirenyatwa Hospital, Harare, Zimbabwe; 123 Pla Director d’oncologia L’Hospitalet de Llobregat, Llobregat, Spain; 124 Princess Margaret Hospital, Nassau, Bahamas; 125 Programa Nacional del Cáncer, Montevideo, Uruguay; 126 Public Health Agency, Quality Assurance Reference Centre, Northern Ireland Cancer Screening Programmes, Belfast, Northern Ireland; 127 Public Health England Screening, London, UK; 128 Public Health Wales, Cardiff, Wales; 129 Public Health England, London, UK; 130 Regionalt Cancer Centrum Stockholm-Gotland, Stockholm, Sweden; 131 Registro Tumori del Veneto, Venice, Italy; 132 Riga East University Hospital, University of Latvia, Riga, Latvia; 133 Riga Stradins University, Riga, Latvia; 134 Rwanda Biomedical Centre, Kigali, Rwanda; 135 Santé Publique France Saint-Maurice, Saint-Maurice, France; 136 School of Public Health, Physiotherapy and Sports Science and National Screening Service, Dublin, Ireland; 137 Scientific Institute of Public Health, Brussels, Belgium; 138 Secretaria de Salud, Tegucigalpa, Honduras; 139 Secretaría de Salud, Mexico City, Mexico; 140 Saint Elisabeth Cancer Institute, Bratislava, Slovak Republic; 141 Statens Serum Institut, Copenhagen, Denmark; 142 Sundhedsdatastyrelsen, Copenhagen, Denmark; 143 Swedish Cervical Screening Registry, Stockholm, Sweden; 144 Tata Trusts (Alamelu Charitable Foundation), Vijayawada, India; 145 Tianjin Medical University Cancer Institute & Hospital, Tianjin, China; 146 Tripoli Cancer Center, Tripoli, Libya; 147 Université Libre de Bruxelles, Brussels, Belgium; 148 University of Antwerp, Antwerp, Belgium; 149 University of Barcelona, Barcelona, Spain; 150 University of Ghent, Ghent, Belgium; 151 Faculty of Medicine and Health, University of Zimbabwe, Harare, Zimbabwe; 152 Vienna, Austria; 153 Dhaka, Bangladesh; 154 Havana, Cuba; 155 Santo Domingo, Dominican Republic; 156 Valletta, Malta; 157 Basseterre, Saint Kitts and Nevis; 158 Kingstown, Saint Vincent and the Grenadines

**Keywords:** Population screening, Health care

## Abstract

The CanScreen5 project is a global cancer screening data repository that aims to report the status and performance of breast, cervical and colorectal cancer screening programs using a harmonized set of criteria and indicators. Data collected mainly from the Ministry of Health in each country underwent quality validation and ultimately became publicly available through a Web-based portal. Until September 2022, 84 participating countries reported data for breast (*n* = 57), cervical (*n* = 75) or colorectal (*n* = 51) cancer screening programs in the repository. Substantial heterogeneity was observed regarding program organization and performance. Reported screening coverage ranged from 1.7% (Bangladesh) to 85.5% (England, United Kingdom) for breast cancer, from 2.1% (Côte d’Ivoire) to 86.3% (Sweden) for cervical cancer, and from 0.6% (Hungary) to 64.5% (the Netherlands) for colorectal cancer screening programs. Large variability was observed regarding compliance to further assessment of screening programs and detection rates reported for precancers and cancers. A concern is lack of data to estimate performance indicators across the screening continuum. This underscores the need for programs to incorporate quality assurance protocols supported by robust information systems. Program organization requires improvement in resource-limited settings, where screening is likely to be resource-stratified and tailored to country-specific situations.

## Abstract (web summary)

In the first results from an ongoing global cancer screening data repository, screening program organization was better overall in Europe compared to other continents; however, there were substantial gaps in implementation across both high- and low-resource settings.

## Main

A decline in cancer-specific mortality can be achieved through the implementation of screening programs for specific cancers; such programs need effective planning, adequate financial, human and technical resources, and stringent quality control^1^. Following the experiences of high-income countries (HICs), several low- and middle-income countries (LMICs) have included cancer screening programs in their national cancer control plans^2^. Many such screening programs, most being in LMICs and some even in HICs, have failed to deliver the expected clinical benefits^3,4^. One of the key factors contributing to the ineffective nature of these programs is the absence of an information system to collect performance data across the screening continuum, from the identification of the target population to the treatment and follow-up of screen-detected cancers and precursor lesions, and using the same for quality improvement of the program. An organization collecting individual-level data of the population offered cancer screening using an information system, and using the same for program management is known as a screening registry.

Many European Union (EU) Member States have remained at the forefront of implementing quality-assured, population-based cancer screening programs with a strong political commitment and adequate resource allocation. These programs are guided mostly by evidence-based recommendations from European quality assurance guidelines in breast, cervical and colorectal cancer (CRC) screening, which consistently highlight the necessity of regular monitoring and evaluation^5–7^. To achieve this, a cancer screening registry is vital to collect, use and store cancer screening data at the individual level that underpins the entire continuum of cancer screening. A screening registry is also an essential tool to implement invitation-based screening and track screen-positive individuals to ensure their compliance to further management.

The International Agency for Research on Cancer (IARC) reported the status and the performance of cancer screening programs from EU Member States in the years 2008 and 2017 (refs. ^8,9^). Such consecutive evaluations permit comparisons in the performance of screening programs using a harmonized set of indicators. Outside the EU, cancer screening evaluation reports have only been published regularly in a few countries^10,11^. Most LMICs have only been reporting screening coverage based on population surveys because of logistic, fiscal and organizational challenges of data collection across the screening continuum^12,13^.

In 2019, IARC launched the Cancer Screening in Five Continents (CanScreen5) project, which aims to collect, analyze and disseminate information on cancer screening programs globally, and encourage and support countries to routinely collect screening performance data. This global project gathers information and performance data on breast, cervical and CRC screening programs in a standardized manner using an online portal (https://canscreen5.iarc.fr/). Validated data made publicly available through the portal will support program managers in cancer screening evaluation, benchmarking, quality improvement and informed policy formulation. This study describes how the CanScreen5 project works and reports the status, organization and performance of screening programs that have participated in the project up to September 2022. The key outcomes of the current study and their implications to inform policies in cancer screening are displayed in Table 1.

**Table 1:** Policy summary and key outcomes

Background	Quality improvement supported by continuous monitoring and evaluation of performance is vital for cancer screening programs. However, there is a lack of global data to evaluate cancer screening implementation in the real world, especially data from LMICs. This prevents such programs from setting their own benchmarks and compare performance with similar regional programs. IARC initiated the CanScreen5 project, a global cancer screening data repository, to fill this gap.
Main findings and limitations	Eighty-four countries joined the CanScreen5 project and shared qualitative or quantitative (or both) data on breast cancer, cervical cancer and CRC screening programs. Substantial heterogeneity exists between cancer sites and between countries regarding program organization, covering screening policies, protocols, governance, financing mechanisms, systems of invitation and recall, processing of data collected for program monitoring and quality assurance. Overall, organization was better in cancer screening programs from Europe than other continents. For specific cancer sites, CRC screening programs showed better organization. Similarly, considerable heterogeneity existed in program performance as noted through the estimation of harmonized indicators. Examination coverage ranged from 1.7% (Bangladesh) to 85.5% (England, United Kingdom) for breast cancer, from 2.1% (Côte d’Ivoire) to 86.3% (Sweden) for cervical cancer, and from 0.6% (Hungary) to 64.5% (the Netherlands) for CRC screening programs. The proportion advised further assessment following a screening test ranged from 0.6% (Chile) to 14.4% (Republic of Korea) for mammography-based screening and from 1.0% (Mozambique) to 2.8% (Bangladesh) for clinical breast examination-based breast cancer screening programs; from 0.5% (Sri Lanka) to 7.8% for cytology (Uruguay); from 2.3% (Kenya) to 78.8% (Ethiopia) for VIA-based cervical screening programs; and from 2.3% (Calvados, France) to 27.2% (Uruguay) for FIT-based CRC screening programs. Regardless of the screening protocol, further assessment participation rates varied substantially across cancer sites and countries, ranging from 39.7% in Morocco to 100% in the Czech Republic, Denmark and Portugal for breast cancer, from 39.0% in Poland to 100% in Hungary for cervical cancer, and from 33.0% in the Republic of Korea to 97.6% in the Czech Republic for CRC screening programs. The high variability in screening test and protocol, test positivity and further assessment compliance was reflected in the precancer and cancer detection rates.
Policy implications	Substantial heterogeneity in screening program performance revealed by the first batch of data from the CanScreen5 project underscores the need for many such programs to do further in-depth analysis of their performance, identify the scope for improvement and take appropriate measures. To implement corrective actions, program managers need to be aware of the implications of the outcome indicators; for example, a low detection rate may indicate poor performance of the screening or diagnostic tests (or both) or low compliance of screen-positives, but may also be due to low prevalence of disease especially in a frequently screened population. The gap in data collection across the screening continuum in both high- and low-resource settings is a concern. Programs need to build robust information systems to be able to capture screening performance data and use the same for quality improvement. Almost all countries worldwide have invested greatly to strengthen disease surveillance mechanisms (including improvement of health information systems) to mitigate the COVID disease pandemic. Cancer screening programs need to leverage these new developments to improve their own performance and quality.

## Results

**Fig. 1:**
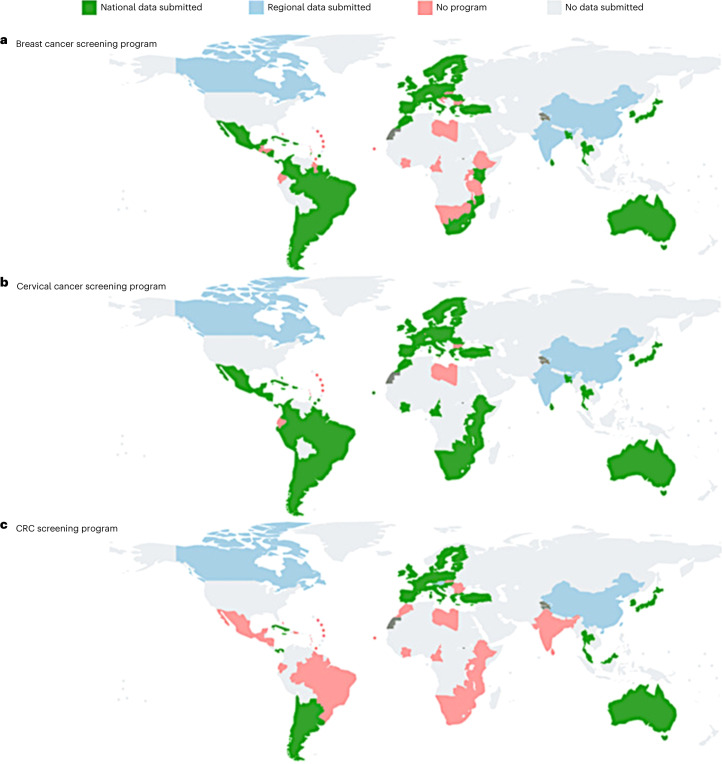
The status of data collection for the CanScreen5 project from various countries for breast cancer, cervical cancer and CRC screening programs. **a**–**c**, Status of data collection for breast cancer (**a**), cervical cancer (**b**) and CRC (**c**) screening. The dotted and dashed lines on the maps represent approximate borderlines for which there may not be full agreement as yet. The designations used and the presentation of the material in this publication do not imply the expression of any opinion whatsoever on the part of the WHO/IARC concerning the legal status of any country, territory, city or area or of its authorities, or concerning the delimitation of its frontiers or boundaries. Map disclaimer: all rights reserved.

Up to September 2022, a total of 84 countries from 5 continents have participated in the CanScreen5 project, including 17 countries from Africa, 27 from the Americas, 10 from Asia, 29 from Europe and 1 from Oceania (Australia). Among these countries, seven (Antigua and Barbuda, Bulgaria, Dominica, Ecuador, Libya, Saint Kitts and Nevis and Saint Lucia) were not included in the analysis because they did not fulfill the minimum criteria of having a screening program for the cancer sites they submitted information on. Fifty-seven countries reported for breast cancer, 75 for cervical cancer and 51 for CRC screening programs. Most of the countries (88.1%, *n* = 74) reported for national programs, while others (Canada, China and India) reported only one or more regional programs (Fig. 1a–c).

### Breast cancer screening programs

Data were obtained from 57 breast cancer screening programs (including regional ones), 4 from Africa, 16 from the Americas, 9 from Asia, 27 from Europe and 1 from Oceania (Australia). Extended Data Tables 1 and 2 provide a summary of the qualitative information on breast cancer screening programs by continent.

### Policies, protocol and organization

Almost half of the European programs (*n* = 12, 44.4%) have a law mandating the government to provide a national breast cancer screening program; however, there were fewer such programs in the Americas (*n* = 3, 18.8%) and Asia (*n* = 2, 22.2%), and none in Africa or Australia. Most programs started in 2000 or later and reported having an individual or a team responsible for implementing the screening activities. While 87.7% of programs provided free-of-charge screening services across the continents (Africa (*n* = 3, 75.0%), Americas (*n* = 13, 81.3%), Asia (*n* = 8, 88.9%), Europe (*n* = 25, 92.6%) and Australia), fewer (64.9%) provided free-of-charge diagnostic services (Africa (*n* = 1, 25.0%), Americas (*n* = 9, 56.3%), Asia (*n* = 5, 55.6%), Europe (*n* = 22, 81.5%) and Australia).

Mammography was the screening test in all European programs, in Australia and in the Americas (*n* = 15, 93.8%); however, only one-third of programs in Asia (*n* = 3, 33.3%) adopted mammography as the screening test. Double reading of mammograms was widely practiced in Europe and Australia but not in the Americas. Clinical breast examination (CBE) was the primary screening test in all African programs.

Invitation to eligible women to participate in screening was reported by 96.3% (*n* = 26) of European programs; however, only 12.5% of programs in the Americas and no programs in Africa did so. Active tracking of screening-positive women to ensure their compliance was performed in screening programs from Africa (*n* = 2, 50%), the Americas (*n* = 10, 62.5%), Asia (*n* = 6, 66.7%), Europe (*n* = 23, 85.2%) and Australia. Most of the programs in Europe (*n* = 24, 88.9%), Asia (*n* = 7, 77.8%), the Americas (*n* = 9, 56.3%) and Australia, but only 1 program in Africa (25%), reported data collection on an individual basis. Likewise, the capability of the programs to link with population-based cancer registries (PBCRs) was highly variable across the continents. Heterogeneity also existed in the manner of quality assurance.

### Quantitative performance data

**Fig. 2:**
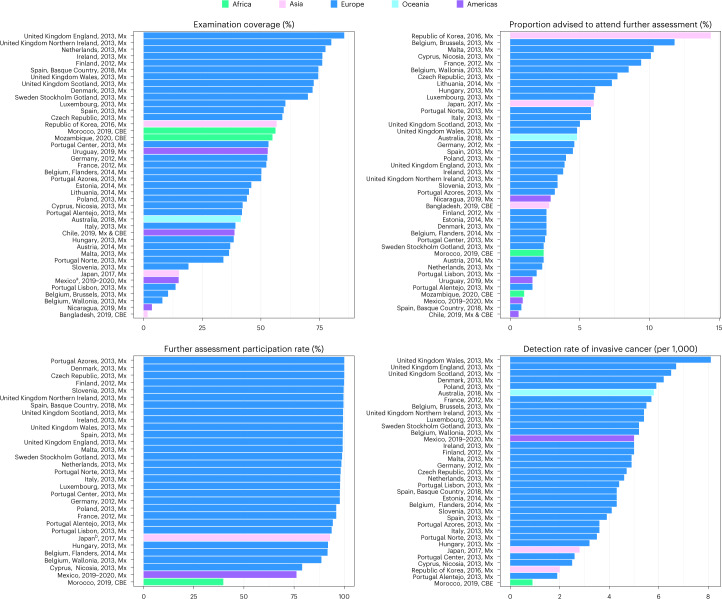
Comparative values of selected performance indicators for the breast cancer screening programs that provided data to the CanScreen5 project. The reporting format is country or region, reporting year and screening protocol. Mx, mammography. ^a^Mexico: the target population is not the total of individuals eligible for screening (age-based). ^b^Japan: women with a negative screening test receiving further assessment due to clinical recommendation were also included.

A comparative analysis of quantitative data on performance collected from 42 breast cancer screening programs (including regional ones) is presented in Fig. 2. Details of the data according to each country can be found in Supplementary Table 1. Screening examination coverage was highly heterogeneous, with reported coverage ranging from 1.7% in Bangladesh to 85.5% in England. Considerable heterogeneity was also observed for the proportion of women put forward for further assessment, ranging from 0.6% (Chile) to 14.4% (Republic of Korea) for mammography-based screening and 1.0% (Mozambique) to 2.8% (Bangladesh) for CBE-based protocols. Furthermore, the assessment participation rate exceeded 90% in most European countries (except in Nicosia (Cyprus) and Wallonia (Belgium)) and Japan, while the rate was only 39.7% in Morocco. For programs adopting mammography as the screening method, the detection rate of carcinoma in situ of the breast ranged from 0.1 per 1,000 (Estonia and Poland) to 2.1 per 1,000 (Wales, United Kingdom); the detection rate for invasive cancer ranged from 1.9 per 1,000 (Portugal) to 8.1 per 1,000 (Wales, United Kingdom). Morocco was the only country with a CBE-based program that provided data on invasive cancers detected; the detection rate of invasive cancer was 0.9 per 1,000.

### Analysis according to World Bank income status

We also analyzed the results according to the World Bank income classification of countries (Supplementary Table 5). HICs had better organized screening programs compared to LMICs, which was supported by a law mandating screening provision in 41.2% (*n* = 14) of them. Compared to LMICs, HICs were also more likely to have an invitation system in place (*n* = 29, 85.3%) and use mammography as the primary screening test (*n* = 33, 97.1%), with double reading of all mammograms (*n* = 23, 67.6%); 85.3% of HICs (*n* = 29) had an information system that collects individual data.

### Cervical cancer screening programs

Seventy-five cervical screening programs (including regional ones) reported data for the CanScreen5 project, 16 from Africa, 22 from the Americas, 9 from Asia, 27 from Europe and 1 from Oceania (Australia). Extended Data Tables 3 and 4 provide a summary of the qualitative information on 75 cervical screening programs by continent.

### Policies, protocol and organization

Policies for cervical screening were mandated by law in 16% (*n* = 12) of the programs (Americas (*n* = 3, 13.6%), Asia (*n* = 2, 22.2%), Europe (*n* = 7, 25.9%)). Screening programs started before 2000 in the Americas (*n* = 9, 40.9%), Europe (*n* = 9, 33.3%), Asia (*n* = 2, 22.2%), Africa (*n* = 1, 6.3%) and Australia. Cervical screening services were provided free of charge in 88% of the programs in the project (Africa (*n* = 13, 81.3%), Americas (*n* = 19, 86.4%), Asia (*n* = 8, 88.9%), Europe (*n* = 25, 92.6%) and Australia). However, colposcopy and biopsy were only available as payable services in Africa (*n* = 8, 50.0%), the Americas (*n* = 13, 59.1%), Asia (*n* = 5, 55.6%) and Europe (*n* = 21, 77.8%). Screening tests were administered free of charge in Australia. Whether women have to pay for diagnostic tests depends on their insurance coverage.

While cytology was the most frequently used screening test in the Americas (*n* = 21, 95.5%), Asia (*n* = 6, 66.7%) and Europe (*n* = 27, 100%), 93.8% (*n* = 15) of the programs in Africa reported using visual inspection with acetic acid (VIA). Human papillomavirus (HPV)-based screening (with or without cytology) was already introduced in Africa (*n* = 4, 25.0%), the Americas (*n* = 10, 45.5%), Asia (*n* = 2, 22.2%), Europe (*n* = 9, 33.3%) and Australia.

Screening invitation was reported in Europe (*n* = 21, 77.8%), Asia (*n* = 5, 55.6%), the Americas (*n* = 5, 22.7%) and Australia, but not in Africa. Most countries in Africa (*n* = 9, 56.3%) only invited human immunodeficiency virus-positive women for cervical screening. Individual-level data collection has been reported in Africa (*n* = 3; 18.8%), the Americas (*n* = 14, 63.6%), Asia (*n* = 7, 77.8%), Europe 74.1% (*n* = 20, 74.1%) and Australia. A link with PBCR was present in 70.4% (*n* = 19) of European programs. The proportion was much lower in the Americas (*n* = 2, 9.1%) and Asia (*n* = 2, 22.2%), and nonexistent in Africa.

### Quantitative performance data

**Fig. 3:**
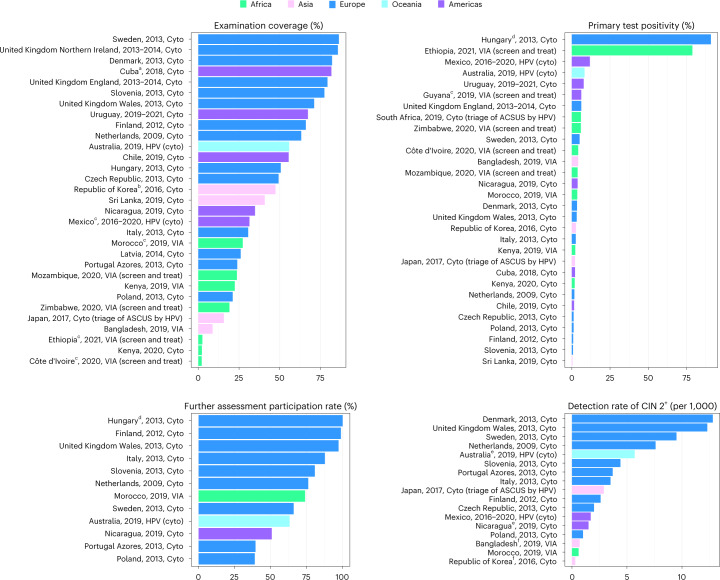
Comparative values of selected performance indicators for cervical cancer screening programs that provided data to the CanScreen5 project. The reporting format is country or region, reporting year and screening protocol. Cyto, cytology. ^a^Cuba: examination coverage might be slightly overestimated because some participants outside the screening program were screened. ^b^Republic of Korea: women with a previous diagnosis of cancer before the examination date were excluded from these screen-related data. ^c^Côte d’Ivoire, Ethiopia, Morocco, Guyana, Mexico: the target population is not the total of individuals eleigible for screening (based on age); the project in Guyana is at the rolling out phase. ^d^Hungary: colposcopy was a substantial part of the screening primary visit. ^e^Nicaragua and Australia: the detection rate was only for CIN 3^+^. ^f^Bangladesh, Republic of Korea: the detection rate was only for cervical cancer.

Only 33 cervical cancer screening programs provided quantitative data. Figure 3 describes a comparative analysis of performance based on those data; details of the data by country are found in Supplementary Table 2. The screening examination coverage ranged from 2.1% in Côte d’Ivoire to 86.3% in Sweden. Substantial heterogeneity existed for screening test positivity rates, ranging from 0.5% in Sri Lanka to 7.8% in Uruguay for cytology, and from 2.3% in Kenya to 78.8% in Ethiopia for VIA. More than half of programs (*n* = 21, 65.6%) could not provide data on participation in further assessment. The estimated participation rate in further assessment ranged from 39.0% in Poland to 98.8% in Finland. Both detection rate and positive predictive value (PPV) could be assessed only for 12 programs providing data on final histopathological diagnosis. For programs using cytology as the primary screening method, the detection rate of CIN 2 or worse lesions (CIN 2^+^) ranged from 1.0 per 1,000 in Poland to 12.8 per 1,000 in Denmark.

Of the four cervical screening programs in Africa and one in South America that adopted a screen-and-treat protocol, only two of these programs provided data on treatment. The treatment rates for screen-positive women were 54.6% in Zimbabwe and 82.3% in Guyana.

### Analysis according to World Bank income status

Unlike breast cancer screening, less variability was observed in the organization of cervical screening programs across countries belonging to different income status. An exception was the availability of diagnostic tests free of charge; while 81.0% (*n* = 17) of upper-middle income countries (UMICs) and 62.9% (*n* = 22) of HICs reported offering free diagnostic tests, only 42,1% (*n* = 8) of LMICs reported offering free diagnostic services. VIA was the primary cervical screening test in 50.0%, 53.8% and 4.8% of LICs, LMICs and UMICs, respectively. None of the HICs reported using VIA.

Like breast cancer screening, cervical cancer screening programs from HICs were more likely than LMICs to have an invitation system in place with an information system collecting individual data (Supplementary Table 6).

### CRC screening programs

Fifty-one CRC screening programs (including regional ones) reported data for the CanScreen5 project: none from Africa; 18 from the Americas; 6 from Asia; 26 from Europe; and 1 from Oceania (Australia). Extended Data Tables 5 and 6 describe the qualitative information from CRC screening programs by continent.

### Policies, protocol and organization

Most programs started in 2000 or later (*n* = 45, 88.2%) and used the fecal immunochemical test (FIT) for screening (*n* = 40, 78.4%). Colonoscopy was used as a primary screening test in Austria, Belgium, the Czech Republic, China, Germany, Greece, Turkey and Poland. The CRC screening programs were better organized than the breast or cervical cancer screening programs, with a high proportion of the programs having a dedicated budget (Americas (*n* = 13, 72.2%), Asia (*n* = 6, 100%), Europe (*n* = 24, 92.3%) and Australia) and provided free-of-charge screening services (Americas (*n* = 16, 88.9%), Asia (*n* = 5, 83.3%), Europe (*n* = 25, 96.2%) and Australia) and diagnostic services (Americas (*n* = 13, 72.2%), Asia (*n* = 4, 83.3%), Europe (*n* = 20, 76.9%) and Australia). Screening invitation was reported by programs in the Americas (*n* = 8, 44.4%), Asia (*n* = 2, 33.3%), Europe (*n* = 23, 88.5%) and Australia; 78.4% (*n* = 40) of the countries and regions collected individual-level screening data. Programs reporting to have links with PBCR by region were Europe (*n* = 16, 61.5%), Asia (*n* = 2, 33.3%), the Americas (*n* = 6, 33.3%) and Australia.

### Quantitative performance data

**Fig. 4:**
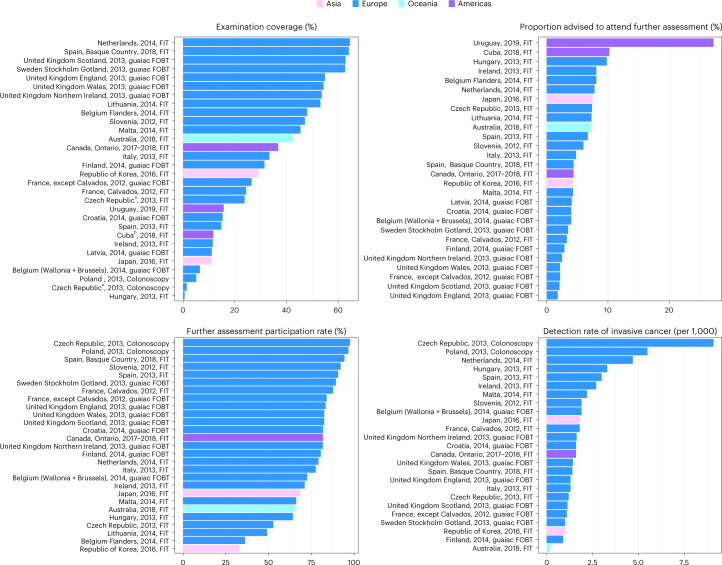
Comparative values of selected performance indicators for CRC screening programs that provided data to the CanScreen5 project. The reporting format is country or region, reporting year and screening protocol. ^a^Czech Republic: examination coverage is underestimated in program-specific age ranges because screened persons aged over 79 are not reported. ^b^Cuba: it was not possible to separate the number of individuals screened opportunistically outside the program, thus coverage may have been overestimated.

Quantitative performance data on CRC screening was submitted by 30 programs; a comparative analysis of the key performance indicators (KPIs) is shown in Fig. 4. Data organized according to country can be found in Supplementary Table 3. Examination coverage ranged from 0.6% in Hungary to 64.5% in the Netherlands. Considerable heterogeneity was observed for screen positivity, ranging from 3.3% in France (Calvados) to 27.2% in Uruguay for FIT-based screening and from 1.8% in England (United Kingdom) to 4.1% in Latvia for guaiac fecal occult blood test (gFOBT). Further assessment participation rate ranged from 33.0% in the Republic of Korea to 97.6% in the Czech Republic. The detection rate for advanced adenoma ranged from 0.8 per 1,000 in Scotland (United Kingdom) to 80.8 per 1,000 in the Czech Republic. The detection rate of invasive cancer ranged from 0.2 per 1,000 in Australia to 9.1 per 1,000 in the Czech Republic. The Czech Republic had colonoscopy-based screening and reported the highest detection rates for both advanced adenoma and CRC.

### Analysis according to World Bank income status

Only UMICs (*n* = 8) and HICs (*n* = 43) had CRC screening programs; the qualitative indicators were similar between countries. More details according to the country or region stratified by income classification are available in Supplementary Table 7.

## Discussion

Screening for breast cancer, cervical cancer and CRC linked with high-quality diagnostic and treatment services demonstrated significant reduction in mortality in randomized controlled trials and ecological studies nested in real-world programs^14–16^. Quality assurance, defined as the process of organizing services within a health program to ensure that the outcomes meet established standards and that health benefits to the target population are maximized, is a key component of screening program organization. An expert group led by IARC in 2022 listed 16 criteria that need to be fulfilled for a screening program to be considered as well organized; these include quality assurance along with policy commitment, screening invitation, information system, screening protocol and call–recall system^17^. Through fulfillment of these criteria, screening programs can ensure that any inherent harms are minimized and outweighed by the potential benefits at the population level.

With an ultimate objective to improve the quality and impact of cancer screening programs, the CanScreen5 project engaged directly with screening program managers and coordinators and trained them to submit information from their own programs related to 13 of the 16 essential criteria identified by the IARC expert group (Methods). With the help of global experts, CanScreen5 also listed and defined key indicators to measure performance across the screening continuum (Supplementary Table 4). Trained participants were requested to submit data from their respective programs to enable the project to estimate these indicators. By triangulating qualitative and quantitative information, strengths and deficiencies can be identified from these programs and the values of performance indicators can be interpreted in the right context. Countries need to learn from each other to adopt best practices and correct internal deficiencies.

A public screening policy formulated through a legislative process is the strongest commitment from the government, ensuring sustained allocation of funds for screening programs^18^. Although mostly reported from HICs in Europe, such good practices have also been reported by countries outside Europe. The Turkish cervical screening program was launched in 2004 with a law mandating that the government must dedicate funding to provide free-of-charge HPV detection-based screening and diagnostic services. The Turkish program complied with many essential criteria of organized screening, such as invitation via text messages or phone calls (or both), active tracking of screen-positive women, a health information system collecting screening-related results and a team responsible for monitoring the program with prespecified performance indicators. In contrast, absence of a strong policy commitment and lack of assured financing (either directly or through insurance coverage) restricted the ability of many screening programs in Africa and Latin America to provide free-of-charge screening and downstream services. Consequentially, these programs suffered from low coverage, low compliance to follow-up and lack of desired impact on cancer burden. Starting in the 1980s, highly organized CRC screening programs have been implemented in some EU countries such as Italy, the Netherlands and Spain with appropriate policy, coordination, financing, screening protocol and invitation, call–recall system and quality assurance. By disseminating such good practice, CanScreen5 provides an opportunity for other programs to improve the organization and quality of their own services.

The most frequently reported performance indicator for screening programs is screening coverage. Ideally, a screening program should be population-based, indicating that the program is capable of identifying screen-eligible individuals and systematically inviting them to participate in screening. Compared to opportunistic screening, population-based screening can achieve higher coverage and ensures more equitable use of resources and higher effectiveness at the population level^19^. These contrasts are seen in breast cancer screening programs in the Republic of Korea and Japan, the former being a population-based one whereas the latter is opportunistic. The screening coverage reported from the Republic of Korea was 56.7% whereas the same from Japan was only 15.1%. However, having a system of invitation alone will not have the desired benefits unless downstream diagnostic and treatment services are adequately strengthened^20^.

The participation rate of screen-positive individuals to further assessment is a very important process indicator to monitor the quality of services and depends on whether a system of active tracking of screen-positive individuals is in place or not. The cervical screening program in Finland, with an active tracking system, achieved a further assessment participation rate of 98.8%; in the Netherlands, the rate was 76.1% without such facilities in place. Despite its high importance, our results show that most of the programs do not collect data to measure this indicator.

Measuring the detection rates of precancer or cancer (or both) is essential as an outcome indicator. The detection rate is impacted by several factors, which may not always be related to the quality of services offered. For example, the highly variable detection rates of CRC in Europe (ranging from 0.9 per 1,000 in Finland to 9.1 per 1,000 in the Czech Republic) could be attributed to differences in the screening test, the positivity cutoff value for FIT (range 15–180 μg g^−1^ feces) or CRC risk of the population, which is impacted by age, sex and screening prevalence. However, quality issues like further assessment compliance, which ranges from 36.3% in Belgium (Flanders) to 97.6% in the Czech Republic in Europe, and quality of diagnostic evaluation could also be responsible for low detection rates. In some instances, the gap in data quality was obvious from the indicators; for example, the detection rate of CIN 2^+^ in Slovenia was four times higher than that reported from Poland, despite being neighboring countries. The most likely explanation for this observation was the difference in compliance to further assessment, which was 80.7% in Slovenia and only 39.0% in Poland.

Collecting data and measuring indicators will be of value only when these are compared to expected standards, usually described as acceptable and desirable, which are often program-specific. Setting standards for the key indicators is an essential requirement for quality assurance, although this may be challenging. The performance standards for mammography screening were developed by the Breast Cancer Surveillance Consortium, including over 2 million screening mammography studies performed in over 1 million women in the United States of America, which indicated that the mean cancer detection rate and mean PPV to detect cancers are 4.7 per 1,000 and 4.8%, respectively^21^. The standards used in the United States of America are higher than those of more than half of the mammography programs in our study, indicating the need for setting at least some regional standards. Currently, European programs have standards for a limited number of indicators, for example, for CRC screening, participation rate to screening out of those invited, further assessment participation rate and completion rate of follow-up colonoscopy^9^. Collecting high-quality data on a continued basis will allow programs to set their own standards.

CanScreen5 also identified irrational policies and cancer screening practices in some countries. A decision to introduce a new screening program depends on disease burden, availability of resources, health system preparedness and prioritization of healthcare needs in the country. LMICs struggling to maintain a cervical cancer screening coverage over 10% have little justification to introduce a breast cancer screening program, as reported from Bangladesh. Kenya and South Africa continue to practice cytology-based screening despite the strong recommendation from the World Health Organization (WHO) to switch to HPV detection or VIA-based screening in settings where quality-assured cytology is difficult to implement^22^. Irrational practices have also been observed in HICs, for example, using colposcopy as a cervical cancer screening tool in Hungary.

An issue of concern is that many programs in our study did not have adequate quantitative data to be able to estimate the KPIs and evaluate their own quality. Among the participating programs, most could not share data beyond the number of participants screened and screen positivity. Lack of data related to further assessment of screen-positive individuals and detection of precancers or cancers may be due to logistic and financial constraints, low prioritization of quality assurance, lack of functioning information systems or hesitancy to publish ‘official’ data. Instead of collecting data from the program, LMICs mostly use data from the WHO’s STEPwise approach to surveillance surveys to report screening coverage^13,23^. Such data are dependent on self-reports, which are subject to recall bias^24^. Moreover, screening coverage correlates poorly with the impact of screening (such as reduction in mortality) as demonstrated in several Latin American countries, underscoring the need for programs to measure the performance of diagnostic and treatment services^25^. Building on the experience from the Canscreen5 project, cancer screening programs should consider the following measures to improve data quality and completeness: (1) conduct a thorough assessment of services associated with screening based on the information and data available; (2) identify the essential criteria for organized programs that are either missing or poorly implemented; (3) develop a feasible, measurable and time-bound plan in consultation with all stakeholders to improve the quality of services at different levels; (4) dedicate an adequate budget for quality assurance and put together a team responsible for implementing quality assurance, if not already in place; (5) build or strengthen information systems to capture performance data so that the quantitative data collection tools can be completed and KPIs can be estimated; (6) create links with population databases (for example, electoral rolls or birth registers) to be able to identify screen-eligible individuals and with PBCR to monitor impact; (7) leverage the vertical investments made to improve surveillance systems and mobile health applications to mitigate the coronavirus disease (COVID) pandemic; and (8) invest in capacity building of policymakers, managers and health professionals engaged in screening-related activities to be able to understand the value and application of quality assurance.

The CanScreen5 project has limitations. Although our ambition was to reach out to all countries and build a data repository as an IARC flagship program, that is, the Global Cancer Observatory (https://gco.iarc.fr), at this stage of the project we could only manage to collect data from a limited number of countries. As the project matures and published data become more visible, we hope to involve more countries as part of the network. The reasons for nonparticipation of some countries we approached to participate include: voluntary nature of participation (no national or global mandate); nonavailability of approval from higher authorities; and reluctance of programs to share data because of the fear of receiving criticism for poor performance. Another limitation is that the data collected from EU countries in 2016 are out of date. A new round of data collection from Europe will be initiated by IARC in 2023 to update these data. Furthermore, the quantitative data from most LMICs is very incomplete. Sometimes programs are reporting the number of examinations and tests performed and not the number of participants undergoing screening, which makes it difficult to exclude participants undergoing repeat testing within a short interval. At this stage, CanScreen5 is collecting screening data on three cancer sites for which screening is most prevalent. However, as screening for other cancer sites becomes evidence-based and is implemented, for example, lung and prostate cancers, we have plans to include these too.

The strength of the CanScreen5 project is that we have not relied on secondary data sources; instead, we collected information provided and validated by program coordinators. This global initiative collects cancer screening performance data beyond screening coverage.

In conclusion, the CanScreen5 project is a dynamic, ongoing activity and not just a one-time data collection project. We will continue with our engagement with countries, especially LMICs, to enhance data collection and quality. Investments in information technology infrastructures, high population coverage with broadband and Internet facilities, and digital capability building of the health workforce to mitigate the COVID pandemic-induced health crisis have created an enabling environment for countries to strengthen multisectoral digital healthcare^26^. We are optimistic that screening programs will take advantage of this accelerated digital transformation to reform the process of data collection. This will in turn improve the quality of data in CanScreen5 and make it an authentic data repository for cancer screening globally.

## Methods

### Overview

The CanScreen5 project was launched in June 2019 and was built on IARC’s successful reporting of the status of implementation and performance of cancer screening programs in EU Member States in collaboration with the Centro di Riferimento per l'Epidemiologia e la Prevenzione Oncologica in Piemonte, Italy and the Finnish Cancer Registry^8,9^. The data collection tools, KPIs and strategies for data collection and validation used in the EU project were further refined to make these tools and strategies globally relevant and suitable for different resource settings. This adaptation was done in consultation with an advisory board consisting of 21 cancer screening experts selected by IARC to represent different geographical regions and healthcare settings.

### Network building and collaboration

CanScreen5 aims to collect information and data directly from each country’s Ministry of Health (MoH). IARC’s existing network of research collaborators across the globe is leveraged to reach out to the MoH. The contact person in the MoH is requested to identify program coordinators or other experts capable of providing reliable information and data. We also liaise with WHO regional offices to establish contact with the MoH. If such contact with the MoH cannot be established in a particular country, we approach the academic or public health institutes (or both) associated with the implementation and evaluation of a screening program to identify potential data providers.

### Training of potential data providers

Identified data providers complete a self-paced virtual learning module. The module describes the objectives of the project, how to collect data using the data collection tools and how to submit the same online to the CanScreen5 portal. The definition of the various performance indicators and how those indicators will be estimated in the project are also explained. The e-learning modules, which are available free of charge on the IARC website (https://learning.iarc.fr/edp/courses/pgm-cancer-screening/), go beyond just describing the methodology of the project. The modules cover principles of cancer screening, planning and implementation of screening programs, and particularly focus on the principles, steps and value of quality assurance in the context of cancer screening programs. Depending on the availability of resources, we organize face-to-face workshops with groups of data providers. Data providers are given password-protected access to the data submission platform after completion of virtual learning.

Participation in the project by the countries was voluntary and no payment was made to the data providers or their staff. We tried to convince the screening program managers to invest in collecting data from their own program budget. This is important for the long-term sustainability of a project of this magnitude.

### Data collection

To be able to submit data to CanScreen5, a country (or a region within the country) should have a ‘screening program’ as per the CanScreen5 definition. The project defines a screening program as one characterized by having at least a formal commitment from the health authorities to provide screening services to a defined eligible population^27^. This commitment must be documented as a law, an official notification or a recommendation. A documented screening protocol and a mechanism of monitoring and supervision are also required to fulfill the criteria of being a screening program.

Data providers can download the qualitative and quantitative data from the project portal, which is available in English, French, Russian and Spanish, to collect information and data from breast, cervical and CRC screening programs separately. Qualitative, freely downloadable data tools available on the portal are used to collect information on screening policies and protocols, governance and financing mechanisms, systems of invitation and recall, process of data collection for program monitoring and protocol for quality assurance. The set of data collection tools includes the corresponding guideline on each item; definitions of key terms are provided (https://canscreen5.iarc.fr/?page=datasources). Screening performance data are collected in quantitative forms across the screening continuum (from invitation to treatment), from national or regional programs. At the time of data submission, the data provider has to specify whether they are reporting for a national or a regional program. During our communications with them, after data submission, we further confirm whether the collected data reflect the entire country or a region only.

The minimum set of quantitative data requested from the programs is the number of individuals screened and the outcomes of the screening tests. Data are not processed further unless this minimum dataset is available from a program.

Data providers are advised to submit the most recent qualitative information on the screening program and quantitative data for any year within the last 5 years. Data may be submitted for multiple consecutive years, if available. Selection of the year(s) for which quantitative data are submitted is at the discretion of the data provider based on the completeness of the data (including follow-up of screen-positive individuals). A data provider who does not have quantitative data may submit only qualitative information related to the program.

### Data quality checks and data validation process

Data submission by any of the data providers to the CanScreen5 data platform triggers a notification to the IARC Secretariat that initiates the internal validation to check for data consistency, completeness and validity. Submitted information is cross-verified with information available from the policy and protocol documents of the programs. The Secretariat tries to resolve any queries or discrepancies through email exchanges and virtual meetings with the data providers. The project has a scientific committee (SC) consisting of 15 international experts in the field of cancer control. The internally validated data from each country are shared with two SC members to be reviewed independently. The data provider is contacted again to resolve any queries from the SC reviewers. Virtual meetings are often organized between the project Secretariat and the team responsible for data collection from a particular screening program to finalize contents based on consensus. The final version of the validated information and data is shared with the data provider for final approval before it is made publicly available and displayed on the CanScreen5 portal. The formats used to display information include fact sheets, data tables, comparison graphs and heatmaps.

### Key definitions

All the quantitative indicators have been clearly defined on the CanScreen5 portal. The numerators and denominators needed to calculate each indicator are described in Supplementary Table 4. The data used for the numerator and denominator to estimate any indicator should be collected over the same time period (a particular year(s) or one round of screening); an individual tested twice during the specified period should be counted only once during that period. Even the terms used in the qualitative data collection form have been clearly defined on the portal to ensure harmonization of data collection. Some of these key definitions are given below.

#### Screening program

A screening program is defined as cancer screening performed in the framework of a publicly mandated program. To be considered a ‘program’ there has to be a commitment from the government to provide the screening services to the eligible population as defined by laws, statutes, regulations or official notifications. In such cases, as a minimum, the eligible population, the screening test and the screening interval should be defined and there should be some mechanism for monitoring and supervision.

#### Screening policy

This is a policy for a specific screening program that specifies the government’s commitment to provide screening services and defines the targeted age and sex groups, the geographical area and other eligibility criteria; the screening test and interval; and requirements for payment or co-payment, if applicable. As a minimum, the screening protocol and repeat interval, and the determinants of eligibility for screening are stated.

#### Screening protocol

A screening protocol is a detailed documented plan on how to deliver the screening activities. As a minimum, the screening protocol should include clear information on eligible individuals, target age, screening test, examination intervals, further assessment, referral system and quality assurance.

#### Individual invitation

An individual invitation, by letter, email, text message, phone call, home visit or other method, to eligible individuals in the target population to participate in the screening program is sent by the coordination team, by primary health centers or by general practitioners.

#### Quality assurance

Quality assurance encompasses activities intended to assure and improve quality at all levels of the screening process to maximize benefits and cost-effectiveness while minimizing harms. It includes the assessment or evaluation of quality, the identification of problems or shortcomings in the delivery of care, the design of activities to overcome these deficiencies and follow-up monitoring to ensure the effectiveness of corrective steps. Quality assurance of the screening process requires a robust system of program management and coordination, ensuring that all aspects of the service are performing adequately.

#### Essential criteria for organized screening

The qualitative data collection tools collect data to assess 13 of the following 16 essential criteria identified by an IARC expert group to define organized cancer screening:^17^ (1) the cancer screening program has a protocol or guideline describing at least the target population, screening intervals, screening tests, referral pathway and management of positive cases; (2) there is a system in place for identifying the target population; (3) there is a system in place for inviting eligible individuals for screening; (4) the cancer screening program has a policy framework from the health authorities defining governance structure, financing, and the goals and objectives of the program; (5) performance of the screening program is evaluated with appropriate indicators; (6) the protocol or guideline at least describes monitoring and evaluation; (7) there is a system in place for notifying the results to the screened individuals and informing them about follow-up; (8) there is a system in place for sending a recall notice to noncompliant individuals; (9) the program can be audited; (10) a specified team or organization is responsible for quality assurance and improvement; (11) the performance of the cancer screening program is evaluated, published and widely disseminated on a regular basis; (12) all activities along the screening pathway are planned, coordinated and evaluated through a quality improvement framework (quality assurance); (13) there is an evidence-based protocol or guideline developed in consensus with most stakeholders; (14) an information system exists with appropriate links between population databases, screening information and cancer registries for screening implementation and evaluation; (15) the screening program has a provision for continued training for service providers; (16) the performance of the screening program is evaluated with reference standards for the indicators.The CanScreen5 project was started before we received recommendations from the IARC expert group. Hence, three indicators (6, 9 and 15) were not included in the qualitative questionnaire. These will be added in the next versions of the data collection tools.

### Statistical analysis

For descriptive analysis on qualitative information, proportion (%) was used for each item according to continent (Africa, Asia, the Americas, Europe and Oceania). For performance indicators on quantitative data, examination coverage, proportion put forward for further assessment, further assessment participation rate, detection rate, PPV of the screening test and treatment rate were calculated for each program using the formulas presented in Supplementary Table 4 (using the CanScreen5 website data manager).

### Ethics and inclusion statement

CanScreen5 is a global cancer screening data repository that collects data across the world, including data from LMICs. Researchers from LMICs submitting data are included as authors in the list of CanScreen5 project collaborators. We fully endorse the Nature Portfolio guidance on LMIC authorship and inclusion and we are strongly committed to the inclusion of researchers from LMICs as the CanScreen5 project moves forwards.

The CanScreen5 project is relevant to all participating countries as they provided qualitative or quantitative data (or both) on cervical, breast and CRC screening programs. Quantitative data were aggregated, covering the screening continuum from identification of the eligible population to treatment.

The IARC ethics committee reviewed the project and waived the requirement for any consent for collecting data. Data providers are mandated to ensure that they have the necessary approvals from authorities to share data.

### Reporting summary

Further information on research design is available in the Nature Portfolio Reporting Summary linked to this article.

## Online content

Any methods, additional references, Nature Portfolio reporting summaries, source data, extended data, supplementary information, acknowledgements, peer review information; details of author contributions and competing interests; and statements of data and code availability are available at 10.1038/s41591-023-02315-6.

## Supplementary information

Supplementary Information Supplementary Tables 1–7.

Reporting Summary

## Figures and Tables

**Extended Data Table 1:**
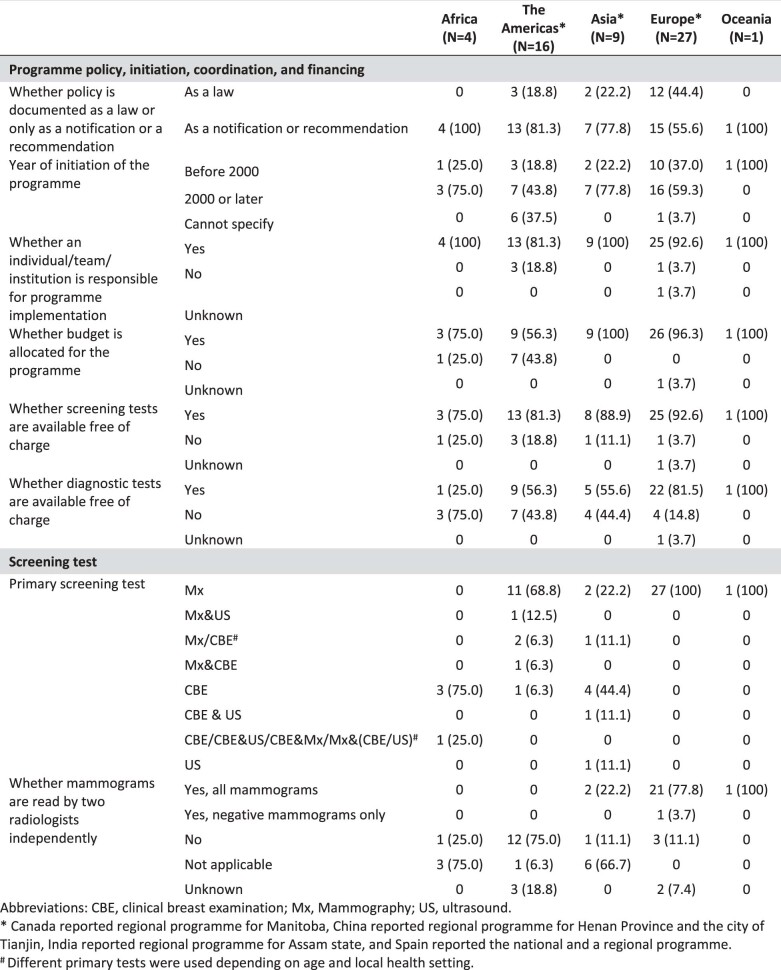
Information on the policy and protocol of breast cancer screening programs by continent

**Extended Data Table 2:**
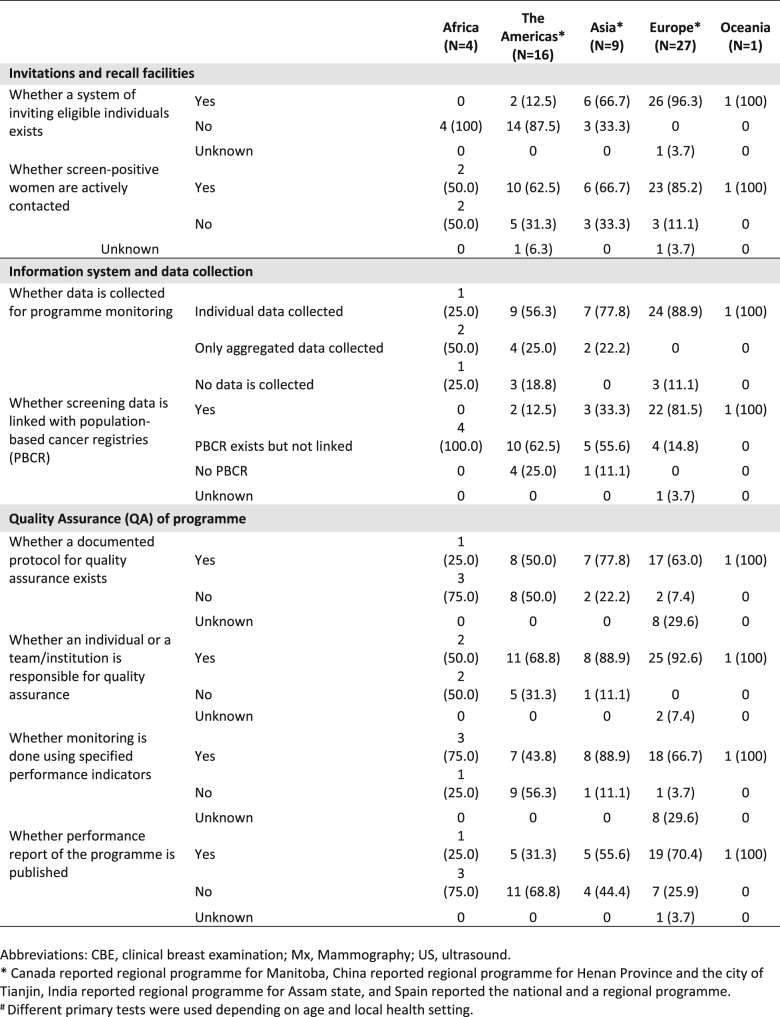
Information on the organization of breast cancer screening programs by continent

**Extended Data Table 3:**
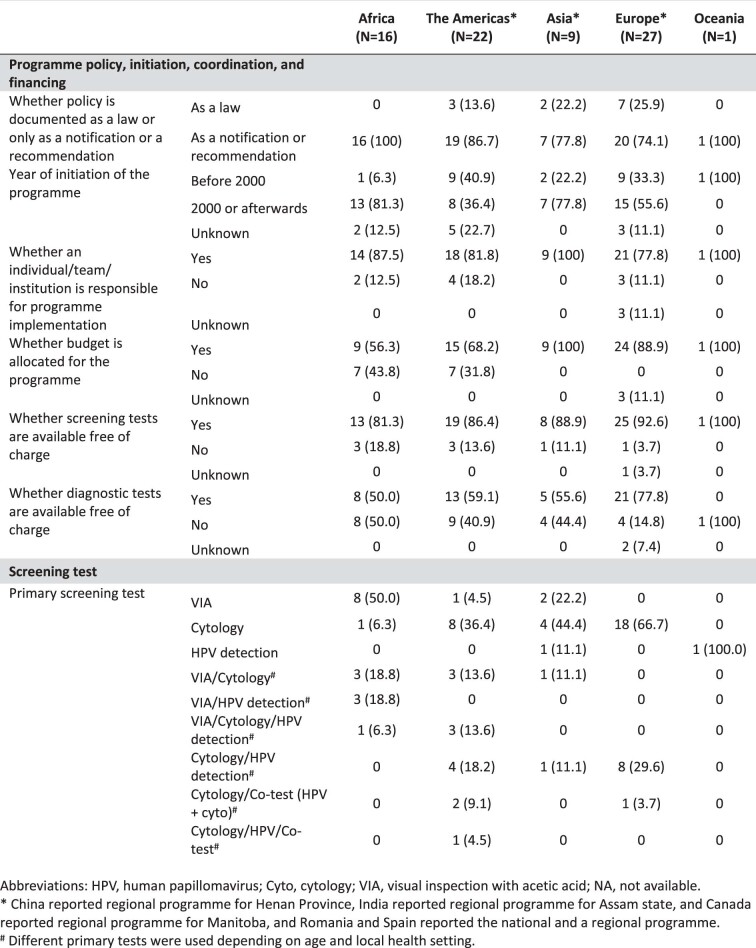
Information on the policies and protocol of cervical cancer screening programs by continent

**Extended Data Table 4:**
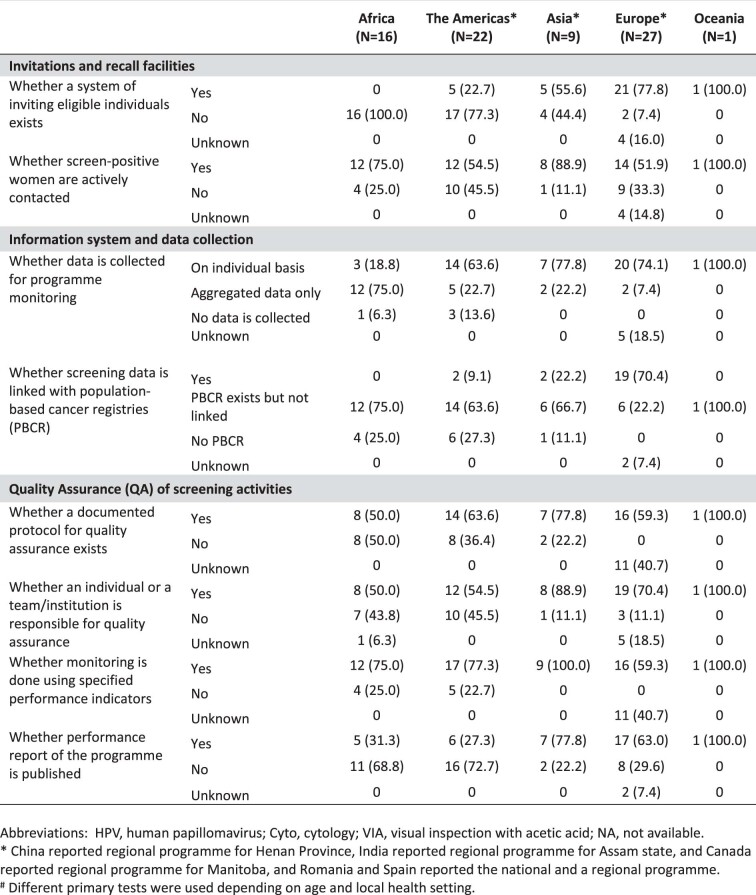
Information on the organization of cervical cancer screening programs by continent

**Extended Data Table 5:**
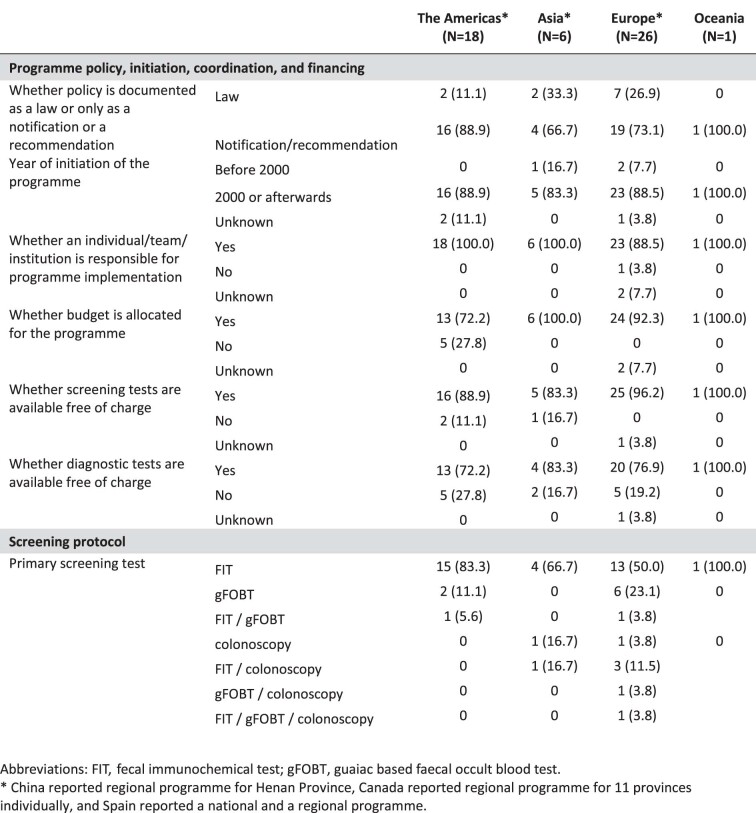
Information on the policies and protocol of colorectal cancer screening programs by continent

**Extended Data Table 6:**
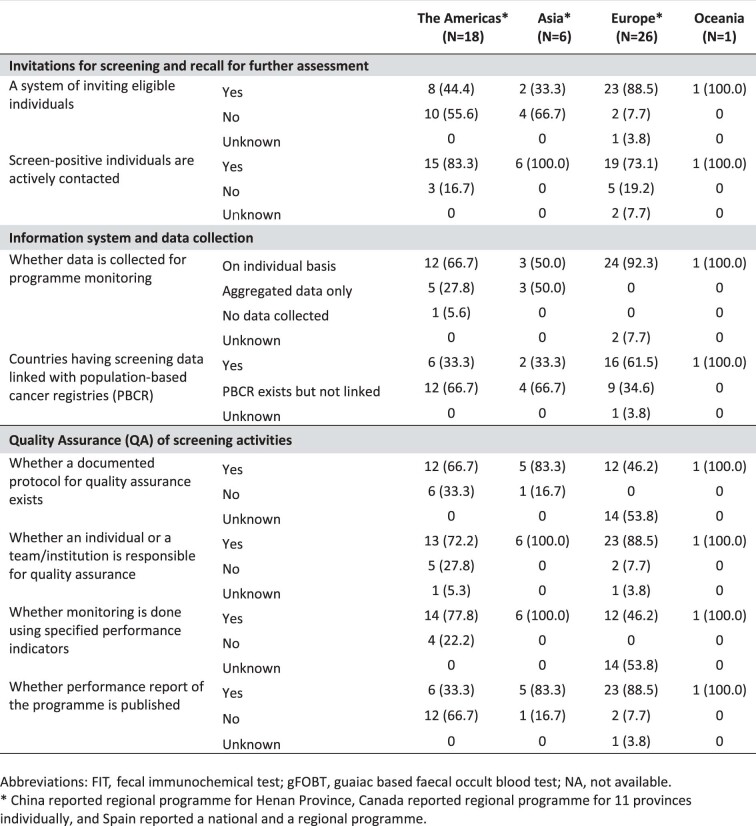
Information on the organization of colorectal cancer screening programs by continent

## Data Availability

The data used in this manuscript are publicly available at https://canscreen5.iarc.fr.

## Code availability

No code was used for data acquisition or analysis.
