# Supplementary material for: CanScreen5, a global repository for breast, cervical and colorectal cancer screening programs
Source: Nat Med. 2023 Apr 27;29(5):1135–45. doi: 10.1038/s41591-023-02315-6 (PMC10202799; doi:10.1038/s41591-023-02315-6)
Supplement: Supplementary file 1 — Supplementary Tables 1–7. [file 41591_2023_2315_MOESM1_ESM.pdf]

nature medicine

Article

https://doi.org/10.1038/s41591-023-02315-6

CanScreen5, a global repository for breast, cervical and colorectal cancer screening programs

In the format provided by the authors and unedited

Supplementary information

Supplementary table S1: Values of selected performance indicators for breast cancer screening programmes providing data to CanScreen5

	Reporting year	Screening test	Screening interval (years)	Age group for which data has been collected (years)	Number of participants screened	Examination coverage (%)	Proportion advised further assessment (%)	Further assessment participation rate (%)	Detection rate of CIS (/1,000)	Detection rate of invasive cancer (/1,000)	PPV to detect CIS & invasive cancer (%)
**Africa**											
Morocco	2019	CBE	2	40-69	1,515,586	56.2	2.4	39.7	NA	0.9	9.7#
Mozambique	2020	CBE	1	25-45	2,453,675	54.9	1.0	NA	NA	NA	NA
**Asia**											
Bangladesh	2019	CBE	1	30+	384,267	1.7	2.8	NA	NA	NA	NA
Japan	2017	Mx	2	40+	3,087,781	15.1	6.0	92.9*	0.4	2.8	5.7
Republic of Korea	2016	Mx	2	40-79	3,712,924	56.7	14.4	NA	0.4	2.0	NA
**The Americas**											
Mexico^	2019-2020	Mx	2	40-69	1,125,869	14.9	0.9	76.2	0.4	5.0	NA
Nicaragua	2019	Mx	1-2	40+	24,218	3.5	2.9	NA	NA	NA	NA
Uruguay	2019	Mx	2	50-69	81,497	52.9	1.6	NA	NA	NA	NA
Chile	2019	Mx and CBE	3	50-69	218,363	38.7	0.6	NA	NA	NA	NA
**Europe**											
Austria	2014	Mx	2	50-69	196,049	36.9	2.4	NA	NA	NA	NA
Belgium, Brussels	2013	Mx	2	50-69	6,040	10.4	11.8	NA	1.0	5.5	9.9

Belgium, Flanders	2014	Mx	2	50-69	204,101	50.2	2.6	91.7	1.0	4.3	22.6
Belgium, Wallonia	2013	Mx	2	50-69	18,063	8.0	8.5	88.6	1.2	5.2	8.5
Cyprus, Nicosia	2013	Mx	2	50-69	8,091	42.2	10.1	79.0	1.0	2.5	4.4
Czech Republic	2013	Mx	2	50-69	418,475	59.1	7.7	100	0.6	4.7	6.8
Denmark	2013	Mx	2	50-69	257,224	72.0	2.6	100	0.6	6.2	27.3
Estonia	2014	Mx	2	50-64	33,106	45.9	2.6	NA	0.1	4.3	NA
Finland	2012	Mx	2	50-69	284.433	76.1	2.6	99.9	0.7	5.0	21.8
France	2012	Mx	2	50-69	2,146,905	52.3	9.4	96.0	1.0	5.7	7.5
Germany	2012	Mx	2	50-69	2,823,631	52.7	4.6	97.8	1.2	4.9	13.5
Hungary	2013	Mx	2	50-64	210,887	38.4	6.1	91.9	0.4	3.2	6.4
Ireland	2013	Mx	2	50-64	144,508	76.2	3.8	99.4	1.3	5.0	16.5
Italy	2013	Mx	1-2	50-69	1,515,395	39.1	5.8	98.0	0.7	3.6	7.6
Lithuania	2014	Mx	2	50-69	93.113	44.9	7.3	NA	NA	NA	NA
Luxembourg	2013	Mx	2	50-69	17,893	60.4	6.0	97.9	1.0	5.4	10.9
Malta	2013	Mx	3	50-69	7,169	36.4	10.3	99.2	1.3	4.9	6.0
Netherlands	2013	Mx	2	50-69	841,444	77.5	2.3	98.6	1.4	4.6	26.3
Poland	2013	Mx	2	50-69	1,141,351	44.0	4.0	96.0	0.1	5.9	15.6
Portugal, Alentejo	2013	Mx	2	50-69	20,589	41.9	1.6	94.3	0.4	1.9	15.0
Portugal, Azores	2013	Mx	2	50-69	7,808	50.1	3.2	100.0	0.3	3.6	11.9

Portugal, Centre	2013	Mx	2	50-69	82,561	53.2	2.5	97.8	0	2.6	10.9
Portugal, Lisboa	2013	Mx	2	50-69	25,960	13.6	1.9	93.7	0.4	4.4	27.9
Portugal, Norte	2013	Mx	2	50-69	82,740	34.0	5.8	98.2	0.7	3.5	7.6
Slovenia	2013	Mx	2	50-69	25,695	19.1	3.4	99.7	2.0	4.1	18.0
Spain	2013	Mx	2	50-69	1,654,865	59.7	4.5	99.3	0.6	3.9	9.3
Spain, Basque Country	2018	Mx	2	50-69	115,420	74.5	0.8	99.6	0.8	4.3	66.2
Sweden Stockholm Gotland	2013	Mx	2	50-69	83,451	70.0	2.4	98.9	0.9	5.2	26.0
UK England	2013	Mx	3	50-70	1,874,528	85.5	3.9	99.2	1.7	6.7	21.6
UK Northern Ireland	2013	Mx	3	50-70	57,110	80.0	3.4	99.6	1.0	5.4	18.7
UK Scotland	2013	Mx	3	50-70	172,427	72.5	5.0	99.4	1.2	6.5	15.4
UK Wales	2013	Mx	3	50-70	101,897	74.4	4.8	99.3	2.1	8.1	21.3
**Oceania**											
Australia	2018	Mx	2	40-74	1,035,907	41.4	4.8	NA	1.5	5.8	NA

Abbreviations: CIS, carcinoma in situ of breast; Mx, mammography; CBE, clinical breast examination; NA, not available; UK, United Kingdom.
Morocco: Only invasive cancer was reported.
^ Mexico: The target population is not the total of age eligible individuals for screening.
*Japan: Women with negative screening test receiving further assessment due to clinical recommendation were also included.

Supplementary table S2: Values of selected performance indicators for cervical screening programmes providing data to CanScreen5

	Reporting period	Screening test (triaging test)	Screening interval (years)	Age group for which data has been collected (years)	Number screened	Examination coverage (%)	Primary test positivity (%)	Further assessment participation rate (%)	Detection rate of CIN2+ (/1000)	PPV of further assessment to detect CIN2+ (%)	Treatment rate (%)
**Africa**											
Côte d'Ivoire*	2020	VIA (screen-and-treat)	3	25-55	45,166	2.1	4.2	NA	NA	NA	NA
Ethiopia*	2021	VIA (screen-and-treat)	3	30-49	12,570	2.5	78.8	NA	NA	NA	NA
Kenya	2020	Cyto	5	25-30, 50-65	28,860	2.2	1.9	NA	NA	NA	NA
Kenya	2019	VIA	5	25-49	300,578	22.4	2.3	NA	NA	NA	NA
Morocco*	2019	VIA	3	30-49	341,476	27.3	3.6	73.9	0.6	2.1	NA
Mozambique	2020	VIA (screen-and-treat)	1	25-54	1,059,470	23.7	3.8	NA	NA	NA	NA
South Africa	2019	Cyto (triage of ACSUS by HPV)	10	30-50	1,025,681	NA	6.0	NA	NA	NA	NA
Zimbabwe	2020	VIA (screen-and-treat)	3	20-49	130,066	19.1	5.9	NA	NA	NA	54.6
**The Americas**											
Cuba[#]	2018	Cyto	3	≤64	866,363	81.7	2.1	NA	NA	NA	NA
Guyana*	2019	VIA (screen-and-treat)	3	25-49	2,931	NA	6.2	NA	NA	NA	82.3
Mexico*	2016-2020	HPV (cyto)	5	35-64	2,839,831	31.4	11.8	NA	1.7	10.0	-
Nicaragua	2019	Cyto	1	20+	690,200	34.8	3.8	50.8	1.5[~]	8.1[¥]	86.8
Uruguay	2019-2021	Cyto	3	21-69	740,021	67.3	7.8	NA	NA	NA	NA
Chile	2019	Cyto	3	25-64	733,630	55.5	1.5	NA	NA	NA	NA

**Asia**											
Bangladesh	2019	VIA	5	30-60	387,719	8.8	4.2	NA	0.7^	NA	NA
Japan	2017	Cyto (triage of ASCUS by HPV)	2	20+	4,294,127	15.7	2.1	NA	2.9	19.1	NA
Republic of Korea	2016	Cyto	2	20-69	4,244,566	47.4[##]	2.7	NA	0.3^	NA	NA
Sri Lanka	2019	Cyto	10	35 and 45	160,938	40.8	0.5	NA	NA	NA	NA
**Europe**											
Czechia	2013	Cyto	3	25-70	2,127,985	49.3	1.2	NA	2.0	NA	NA
Denmark	2013	Cyto	3-5	23-65	402,561	82.1	3.4	NA	12.8	NA	NA
Finland	2012	Cyto	5	30-64	164,878	66.0	0.9	98.8	2.6	29.3	NA
Hungary	2013	Cyto	3	25-65	491,201	50.6	90.9**	100**	NA	NA	NA
Italy	2013	Cyto	3	25-64	1,692,125	30.6	2.6	87.7	3.5	16.0	NA
Latvia	2014	Cyto	3	25-69	55,487	26.0	NA	NA	NA	NA	NA
Netherlands	2009	Cyto	5	30-64	504,338	63.2	1.7	76.1	7.6	59.5	NA
Poland	2013	Cyto	3	25-59	694,719	21.1	1.2	39.0	1.0	23.2	NA
Portugal Azores	2013	Cyto	3	25-64	5,440	23.9	NA	39.5	3.7	40.8	NA
Slovenia	2013	Cyto	3	20-64	164,364	77.4	0.8	80.7	4.4	65.4	NA
Sweden	2013	Cyto	3-5	23-60	629,728	86.3	5.1	66.0	9.5	28.4	NA
UK England	2013-2014	Cyto	3-5	25-64	3,225,180	79.3	6.2	NA	NA	NA	NA

UK Northern Ireland	2013-2014	Cyto	3-5	25-64	118,893	85.7	NA	NA	NA	NA	NA
UK Wales	2013	Cyto	3	25-64	186,430	71.1	3.2	97.2	12.3	39.7	NA
**Oceania**											
Australia	2019	HPV (cyto)	5	25-74	1,886,189	55.8	8.3	63.2	5.72~	48.2¥	NA

Abbreviations: HPV, human papillomavirus; Cyto, cytology; VIA, visual inspection with acetic acid; NA, not available; UK, United Kingdom.
Cuba: Examination coverage might be slightly over estimated, because some participants outside of the screening programme were screened.
*Côte d'Ivoire, Ethiopia, Morocco, Guyana, Mexico: Target population is not the total of age eligible individuals for screening, the project in Guyana is at the phase of rolling-out.
^Bangladesh, Republic of Korea: The detection rate of CIN2+ was only for cervical cancer.
Republic of Korea: Women with a previous diagnosis of cancer prior to the examination date were excluded from this screened-related data.
** Hungary: Colposcopy was substantially part of the screening primary visit.
~ Nicaragua and Australia: The detection rate of CIN2+ was only for CIN3+.
¥ Nicaragua and Australia: PPV for CIN2+ was only for CIN3+.

Supplementary table S3: Values of selected performance indicators for colorectal cancer screening programmes providing data to CanScreen5

	Reporting period	Screening test	Screening Interval (years)	Age group for which data has been collected (years)	Number of participants screened	Examination coverage (%)	Proportion advised further assessment (%)	Further assessment participation rate (%)	Detection rate of adenomas (/1000)	Detection rate of advanced adenomas (/1000)	Detection rate of invasive cancer (/1000)	PPV to detect advanced adenomas (%)	PPV to detect invasive cancer (%)
**The Americas**													
Canada, Ontario	2017-2018	FIT	2	50-74	952,076	36.8	4.4	81.8	NA	NA	1.6	NA	4.7
Uruguay	2019	FIT	2	50-74	55,033	15.7	27.2	NA	NA	NA	NA	NA	NA
Cuba*	2018	FIT	1	50+	465,823	11.7	10.2	NA	NA	NA	NA	NA	NA
**Asia**													
Japan	2016	FIT	1	40+	8,475,677	11.0	7.5	68.6	12.6	1.9	1.8	3.7	3.5
Republic of Korea	2016	FIT	1	50-79	4,790,315	29.2	4.3	33.0	NA	NA	1.0	NA	7.1
**Europe**													
Belgium Flemish	2014	FIT	2	50-74	317,521	48.0	8.1	36.3	NA	NA	NA	NA	NA
Belgium (Wallonia +Brussels)	2014	gFOBT	2	50-74	41,490	6.5	4.0	72.6	11.3	4.6	1.9	15.9	6.7
Croatia	2014	gFOBT	2	50-74	101,476	15.3	4.0	81.9	10.6	NA	1.6	NA	4.9
Czech Republic^	2013	FIT	1-2	50+	535,709	23.8	7.4	52.8	15.3	7.1	1.2	18.2	3.0
Finland	2014	gFOBT	2	60-69	59,250	31.5	2.9	80.6	7.0	0.9	0.9	4.1	3.8
France, except Calvados	2012	gFOBT	2	50-74	2,366,408	26.5	2.2	84.0	6.3	3.7	1.1	19.8	5.6
France, Calvados	2012	FIT	2	50-74	23,795	24.4	3.3	87.8	10.8	6.1	1.8	21.1	6.3
Hungary	2013	FIT	2	50-70	8,318	0.6	9.8	64.2	32.0	NA	3.3	NA	6.1

Italy	2013	FIT	2	50-69	1,981,349	33.4	4.8	77.6	14.2	7.4	1.3	19.7	3.5
Ireland	2013	FIT	2	60-69	23,482	11.5	8.1	71.2	29.2	NA	2.7	NA	4.7
Latvia	2014	gFOBT	1	50-74	68,498	11.1	4.1	NA	NA	NA	NA	NA	NA
Lithuania	2014	FIT	2	50-74	234,257	53.1	7.3	49.2	NA	NA	NA	NA	NA
Malta	2014	FIT	2	60-64	6,754	45.4	4.3	66.2	17.2	NA	2.2	NA	7.8
Netherlands	2014	FIT	2	55-75	529,056	64.5	7.8	79.2	35.6	23.0	4.7	37.9	7.8
Slovenia	2012	FIT	2	50-69	126,327	47.1	6.0	92.2	28.1	17.3	1.9	31.2	3.4
Spain	2013	FIT	2	50-69	449,868	14.8	6.7	90.7	33.4	22.9	3.0	37.8	4.9
Spain, Basque Country	2018	FIT	2	50-69	192,791	64.1	4.4	94.4	8.6	15.1	1.4	36.5	3.3
Sweden Stockholm Gotland	2013	gFOBT	2	60-69	70,410	62.7	3.5	89.4	7.5	3.7	1.0	8.5	3.3
Czech Republic^	2013	Colonoscopy	10	55+	4,504	1.4	NA	97.6	277.3	80.8	9.1	NA	NA
Poland	2013	Colonoscopy	10+	55-64	9,312	5.1	NA	96.9	281.1	57.2	5.5	NA	NA
UK England	2013	gFOBT	2	60-74	2,171,029	54.9	1.8	83.4	5.4	1.5	1.3	10.0	8.6
UK Northern Ireland	2013	gFOBT	2	60-74	66,051	53.6	2.5	81.7	9.5	NA	1.6	NA	8.1
UK Scotland	2013	gFOBT	2	50-74	491,531	62.8	2.1	82.6	6.2	0.8	1.1	5.0	6.9
UK Wales	2013	gFOBT	2	60-74	140,156	54.3	2.2	82.6	4.2	NA	1.4	NA	7.9
**Oceania**													
Australia	2018	FIT	2	50-74	1,075,391	42.4	7.3	65.5	3.0	2.7	0.2	5.6	0.5

Abbreviations: FIT: fecal immunochemical test; gFOBT: guaiac based faecal occult blood test; NA: not available; UK: United Kingdom.
*Cuba: It was not possible to separate number of individuals screened opportunistically outside the programme. Coverage may have been over-estimated.
^Czech Republic: Examination coverage is underestimated in programme-specific age ranges because screened persons aged over 79 years are not reported.

Supplementary table S4: Key performance indicators for cancer screening and their definitions used in CanScreen5 project

**All the performance cancer screening indicators should be calculated for a specific time frame, the defined screening round or the reporting period**
**Examination coverage (%)**<br>$\frac{\text{Number of individuals screened of the eligible population (as defined by the programme protocol) in a specific time frame}}{\text{Number of eligible population (as defined by the programme protocol) in the same specific time frame}} \times 100$
**Proportion advised further assessment (%)**<br>$\frac{\text{Number of individuals with positive test outcome requiring further investigations in a specific time frame}}{\text{Number of participants screened for whom test outcomes are available in the same specific time frame}} \times 100$
**Further assessment participation rate (%)**<br>$\frac{\text{Number of individuals undergone further assessment in a specific time frame}}{\text{Number of individuals with positive test outcome requiring further assessment in the same specific time frame}} \times 100$
**Detection rate (/1,000)**<br>$\frac{\text{Number of individuals with pathologically proven precancer/cancer detected in a specific time frame}}{\text{Number of individuals screened in the same specific time frame}} \times 1{,}000$
**Positive predictive value (PPV) of the screening test (/100)**<br>$\frac{\text{Number of individuals with pathologically proven precancer/cancer detected in a specific time frame}}{\text{Number of screen positive individuals with further assessment performed in the same specific time frame}} \times 100$
**Treatment rate (%)**<br>$\frac{\text{Number of individuals treated for precancer/cancer detected in a specific time frame}}{\text{Number of individuals with pathologically proven precancer/cancer detected in the same specific time frame}} \times 100$

Supplementary table S5: Information on policy, protocol and organisation of breast cancer screening programmes by World Bank income classification of countries

		Low income (N=1)	Lower middle income (N=7)	Upper middle income (N=15)	High income (N=34)
**Programme policy, initiation, coordination, and financing**					
Whether policy is documented as a law or only as a notification or a recommendation	As a law	0	1(14.3)	2(13.3)	14 (41.2)
	As a notification or recommendation	1 (100)	6 (85.7)	13 (86.7)	20 (58.8)
Year of initiation of the programme	Before 2000	0	2 (28.6)	1 (6.7)	14 (41.2)
	2000 or later	1 (100)	4 (57.1)	10 (66.7)	18 (52.9)
	Cannot specify	0	1 (14.3)	4 (26.7)	2 (5.9)
Whether an individual/team/ institution is responsible for programme implementation	Yes	1 (100)	7 (100)	13 (86.7)	31 (91.2)
	No	0	0	2 (13.3)	2 (5.9)
	Unknown	0	0	0	1 (2.9)
Whether budget is allocated for the programme	Yes	1 (100)	6 (85.7)	10 (66.7)	31 (91.2)
	No	0	1 (14.3)	5 (33.3)	2 (5.9)
	Unknown	0	0	0	1 (2.9)
Whether screening tests are available free of charge	Yes	1 (100)	6 (85.7)	12 (80.0)	31 (91.2)
	No	0	1 (14.3)	3 (20.0)	2 (5.9)
	Unknown	0	0	0	1 (2.9)
Whether diagnostic tests are available free of charge	Yes	1 (100)	3 (42.9)	10 (66.7)	24 (70.6)
	No	0	4 (57.1)	5 (33.3)	9 (26.5)
	Unknown	0	0	0	1 (2.9)
**Screening test**					
Primary screening test	Mx	0	2 (28.6)	7 (46.7)	33 (97.1)
	Mx&US	0	0	0	1 (2.9)
	Mx/CBE#	0	0	3 (20.0)	0
	Mx&CBE	0	0	0	0
	CBE	1 (100)	4 (57.1)	3 (20.0)	0
	CBE & US	0	0	1 (6.7)	0
	CBE/CBE&US/CBE&Mx/Mx&(CBE/US)#	0	1 (14.3)	0	0
	US	0	0	1 (6.7)	0
Whether mammograms are read by two radiologists independently	Yes, all mammograms	0	0	1 (6.7)	23 (67.6)
	Yes, negative mammograms only	0	0	0	1 (2.9)
	No	0	3 (42.9)	7 (46.7)	7 (20.6)
	Not applicable	1 (100)	4 (57.1)	5 (33.3)	0
	Unknown	0	0	2 (13.3)	3 (8.8)
**Invitations and recall facilities**					
Whether a system of inviting eligible individuals exists	Yes	0	2 (28.6)	4 (26.7)	29 (85.3)
	No	1 (100)	5 (71.4)	11 (73.3)	4 (11.8)
	Unknown	0	0	0	1 (2.9)
Whether screen-positive women are actively contacted	Yes	1 (100)	4 (57.1)	8 (53.3)	29 (85.3)
	No	0	3 (42.9)	7 (46.7)	3 (8.8)
Unknown		0	0	0	2 (5.9)
**Information system and data collection**					
Whether data is collected for programme monitoring	Individual data collected	0	4 (57.1)	9 (60.0)	29 (85.3)
	Only aggregated data collected	1 (100)	2 (28.6)	3 (20.0)	2 (5.9)
	No data is collected	0	1 (14.3)	3 (20.0)	3 (8.8)
Whether screening data is linked with population-based cancer registries (PBCR)	Yes	0	1 (14.3)	1 (6.7)	26 (76.5)
	PBCR exists but not linked	1 (100)	4 (57.1)	11 (73.3)	7 (20.6)
	No PBCR	0	2 (28.6)	3 (20.0)	0

	Unknown	0	0	0	1 (2.9)
**Quality Assurance (QA) of programme**					
Whether a documented protocol for quality assurance exists	Yes	0	4 (57.1)	8 (53.3)	22 (64.7)
	No	1 (100)	3 (42.9)	7 (46.7)	4 (11.8)
	Unknown	0	0	0	8 (23.5)
Whether an individual or a team/institution is responsible for quality assurance	Yes	1 (100)	5 (71.4)	11 (73.3)	30 (88.2)
	No	0	2 (28.6)	4 (26.7)	2 (5.9)
	Unknown	0	0	0	2 (5.9)
Whether monitoring is done using specified performance indicators	Yes	1 (100)	5 (71.4)	8 (53.3)	23 (67.6)
	No	0	2 (28.6)	7 (46.7)	3 (8.8)
	Unknown	0	0	0	8 (23.5)
Whether performance report of the programme is published	Yes	1 (100)	1 (14.3)	5 (33.3)	24 (70.6)
	No	0	6 (85.7)	10 (66.7)	9 (26.5)
	Unknown	0	0	0	1 (2.9)

CBE: clinical breast examination; Mx: Mammography; US: ultrasound
* Canada reported regional programme for Manitoba, China reported regional programme for Henan Province and the city of Tianjin, India reported regional programme for Assam state, and Spain reported the national and a regional programme.
# Different primary tests used depending on age and local health settings

Supplementary table S6: Information on policy, protocol and organisation of cervical cancer screening programmes by income level

		Low income (N=6)	Lower middle income (N=13)	Upper middle income (N=21)	High income (N=35)
**Programme policy, initiation, coordination, and financing**					
Whether policy is documented as a law or only as a notification or a recommendation	As a law	0	1 (7.7)	2 (9.5)	9 (25.7)
	As a notification or recommendation	6 (100)	12 (92.3)	19 (90.5)	26 (74.3)
Year of initiation of the programme	Before 2000	0	4 (30.8)	4 (19.0)	14 (40.0)
	2000 or afterwards	6 (100)	7 (53.8)	13 (61.9)	17 (48.6)
	Unknown	0	2 (15.4)	4 (19.0)	4 (11.4)
Whether an individual/team/ institution is responsible for programme implementation	Yes	6 (100)	10 (76.9)	19 (90.5)	28 (80.0)
	No	0	3 (23.1)	2 (9.5)	4 (11.4)
	Unknown	0	0	0	3 (8.6)
Whether budget is allocated for the programme	Yes	4 (66.7)	9 (69.2)	15 (71.4)	30 (85.7)
	No	2 (33.3)	4 (30.8)	6 (28.6)	2 (5.7)
	Unknown	0	0	0	3 (8.6)
Whether screening tests are available free of charge	Yes	6 (100)	10 (76.9)	18 (85.7)	32 (91.4)
	No	0	3 (23.1)	3 (14.3)	2 (5.7)
	Unknown	0	0	0	1 (2.9)
Whether diagnostic tests are available free of charge	Yes	3 (50.0)	5 (38.5)	17 (81.0)	22 (62.9)
	No	3 (50.0)	8 (61.5)	4 (19.0)	11 (31.4)
	Unknown	0	0	0	2 (5.7)
**Screening test**					
Primary screening test	VIA	3 (50.0)	7 (53.8)	1 (4.8)	0
	Cytology	0	2 (15.4)	8 (38.1)	22 (62.8)
	HPV detection	0	0	1 (4.8)	1 (2.9)
	VIA/Cytology#	0	2 (15.4)	5 (23.8)	0
	VIA/HPV detection#	3 (50.0)	0	0	0
	VIA/Cytology/HPV detection#	0	0	4 (19.0)	0
	Cytology/HPV detection#	0	2 (15.4)	2 (9.5)	8 (22.9)
	Cytology/Co-test (HPV + cyto)#	0	0	0	3 (8.6)
	Cytology/HPV/Co-test#	0	0	0	1 (2.9)
**Invitations and recall facilities**					
Whether a system of inviting eligible individuals exists	Yes	0	4 (30.8)	4 (19.0)	24 (68.6)
	No	6 (100)	9 (69.2)	17 (81.0)	7 (20)
	Unknown	0	0	0	4 (11.4)
Whether screen-positive women are actively contacted	Yes	6 (100)	9 (69.2)	12 (57.1)	20 (57.1)
	No	0	4 (30.8)	9 (42.9)	11 (31.4)
	Unknown	0	0	0	4 (11.4)
**Information system and data collection**					
Whether data is collected for programme monitoring	On individual basis	2 (33.3)	6 (46.2)	11 (52.4)	26 (74.3)
	Aggregated data only	4 (66.7)	6 (46.2)	8 (38.1)	3 (8.6)
	No data is collected	0	1 (7.7)	2 (9.5)	1 (2.9)
	Unknown	0	0	0	5 (14.3)

		Low income (N=6)	Lower middle income (N=13)	Upper middle income (N=21)	High income (N=35)
Whether screening data is linked with population-based cancer registries (PBCR)	Yes	0	1 (7.7)	0	22 (62.9)
	PBCR exists but not linked	4 (66.7)	8 (61.5)	16 (76.2)	11 (31.4)
	No PBCR	2 (33.3)	4 (30.8)	5 (23.8)	0

	Unknown	0	0	0	2 (5.7)
**Quality Assurance (QA) of screening activities**					
Whether a documented protocol for quality assurance exists	Yes	4 (66.7)	4 (30.8)	16 (76.2)	22 (62.9)
	No	2 (33.3)	9 (69.2)	5 (23.8)	2 (5.7)
	Unknown	0	0	0	11 (31.4)
Whether an individual or a team/institution is responsible for quality assurance	Yes	4 (66.7)	6 (46.2)	13 (61.9)	25 (71.4)
	No	2 (33.3)	7 (53.8)	7 (33.3)	5 (14.3)
	Unknown	0	0	1 (4.8)	5 (14.3)
Whether monitoring is done using specified performance indicators	Yes	5 (83.3)	10 (76.9)	17 (81.0)	23 (65.7)
	No	1 (16.7)	3 (23.1)	4 (19.0)	1 (2.9)
	Unknown	0	0	0	11 (31.4)
Whether performance report of the programme is published	Yes	3 (50.0)	2 (15.4)	8 (38.1)	23 (65.7)
	No	3 (50.0)	11 (84.6)	13 (61.9)	10 (28.6)
	Unknown	0	0	0	2 (5.7)

Abbreviations: HPV, human papillomavirus; Cyto, cytology; VIA, visual inspection with acetic acid; NA, not available.
* China reported regional programme for Henan Province, India reported regional programme for Assam state, and Canada reported regional programme for Manitoba, and Romania and Spain reported the national and a regional programme.
# Different primary tests were used depending on age and local health setting.

Supplementary table S7: Information on policy, protocol and organisation of colorectal cancer screening programmes by income level

		Upper middle income (N=8)	High income (N=43)
**Programme policy, initiation, coordination, and financing**			
Whether policy is documented as a law or only as a notification or a recommendation	Law	2(25.0)	9(20.9)
	Notification/recommendation	6 (75.0)	34 (79.1)
Year of initiation of the programme	Before 2000	0	3 (7.0)
	2000 or afterwards	7 (87.5)	38 (88.4)
	Unknown	1 (12.5)	2 (4.7)
Whether an individual/team/ institution is responsible for programme implementation	Yes	8 (100)	40 (93.0)
	No	0	1 (2.3)
	Unknown	0	2 (4.7)
Whether budget is allocated for the programme	Yes	6 (75.0)	38(88.4)
	No	2 (25.0)	3 (7.0)
	Unknown	0	2 (4.7)
Whether screening tests are available free of charge	Yes	8 (100)	39 (90.7)
	No	0	3 (7.0)
	Unknown	0	1 (2.3)
Whether diagnostic tests are available free of charge	Yes	5 (62.5)	33 (76.7)
	No	3 (37.5)	9 (20.9)
	Unknown	0	1 (2.3)
**Screening protocol**			
Primary screening test	FIT	5 (62.5)	28 (65.1)
	gFOBT	0	8 (18.6)
	FIT / gFOBT	1 (12.5)	1 (2.3)
	colonoscopy	1 (12.5)	1 (2.3)
	FIT / colonoscopy	1 (12.5)	3 (7.0)
	gFOBT / colonoscopy	0	1 (2.3)
	FIT / gFOBT / colonoscopy	0	1 (2.3)
**Invitations for screening and recall for further assessment**			
A system of inviting eligible individuals	Yes	2 (25.0)	32 (74.4)
	No	6 (75.0)	10 (23.3)
	Unknown	0	1 (2.3)
Screen-positive individuals are actively contacted	Yes	7 (87.5)	34 (79.1)
	No	1 (12.5)	7 (16.3)
	Unknown	0	2 (4.7)
**Information system and data collection**			
Whether data is collected for programme monitoring	On individual basis	4 (50.0)	36 (83.7)
	Aggregated data only	4 (50.0)	4 (9.3)
	No data collected	0	1 (2.3)
	Unknown	0	2 (4.7)
Countries having screening data linked with population-based cancer registries (PBCR)	Yes	0	25 (58.1)
	PBCR exists but not linked	8 (100)	17 (39.5)
	Unknown	0	1 (2.3)
**Quality Assurance (QA) of screening activities**			
Whether a documented protocol for quality assurance exists	Yes	5 (62.5)	25 (58.1)
	No	3 (37.5)	4 (9.3)
	Unknown	0	14 (32.6)
	Yes	7 (87.5)	36 (83.7)

Whether an individual or a team/institution is responsible for quality assurance	No	1 (12.5)	6 (14.0)
	Unknown	0	1 (2.3)
Whether monitoring is done using specified performance indicators	Yes	7 (87.5)	26 (60.5)
	No	1 (12.5)	3 (7.0)
	Unknown	0	14 (32.6)
Whether performance report of the programme is published	Yes	3 (37.5)	32 (74.4)
	No	5 (62.5)	10 (23.3)
	Unknown	0	1 (2.3)

Abbreviations: FIT, fecal immunochemical test; gFOBT, guaiac based faecal occult blood test; NA, not available.
* China reported regional programme for Henan Province, Canada reported regional programme for 11 provinces. individually, and Spain reported a national and a regional program.
