# Supplementary material for: CanScreen5, a global repository for breast, cervical and colorectal cancer screening programs
Source: Nat Med. 2023 Apr 27;29(5):1135–45. doi: 10.1038/s41591-023-02315-6 (PMC10202799; doi:10.1038/s41591-023-02315-6)
Supplement: Supplementary file 2 — Reporting Summary [file 41591_2023_2315_MOESM2_ESM.pdf]

nature portfolio

Corresponding author(s): Partha Basu

Last updated by author(s): Feb 15, 2023

Reporting Summary

Nature Portfolio wishes to improve the reproducibility of the work that we publish. This form provides structure for consistency and transparency in reporting. For further information on Nature Portfolio policies, see our Editorial Policies and the Editorial Policy Checklist.

Statistics

For all statistical analyses, confirm that the following items are present in the figure legend, table legend, main text, or Methods section.

n/a	Confirmed	
☐	☒	The exact sample size (*n*) for each experimental group/condition, given as a discrete number and unit of measurement
☒	☐	A statement on whether measurements were taken from distinct samples or whether the same sample was measured repeatedly
☒	☐	The statistical test(s) used AND whether they are one- or two-sided<br>*Only common tests should be described solely by name; describe more complex techniques in the Methods section.*
☒	☐	A description of all covariates tested
☒	☐	A description of any assumptions or corrections, such as tests of normality and adjustment for multiple comparisons
☒	☐	A full description of the statistical parameters including central tendency (e.g. means) or other basic estimates (e.g. regression coefficient) AND variation (e.g. standard deviation) or associated estimates of uncertainty (e.g. confidence intervals)
☒	☐	For null hypothesis testing, the test statistic (e.g. *F*, *t*, *r*) with confidence intervals, effect sizes, degrees of freedom and *P* value noted<br>*Give P values as exact values whenever suitable.*
☒	☐	For Bayesian analysis, information on the choice of priors and Markov chain Monte Carlo settings
☒	☐	For hierarchical and complex designs, identification of the appropriate level for tests and full reporting of outcomes
☒	☐	Estimates of effect sizes (e.g. Cohen's *d*, Pearson's *r*), indicating how they were calculated

Our web collection on statistics for biologists contains articles on many of the points above.

Software and code

Policy information about availability of computer code

Data collection	No commercial software was used in this study to collect data.
Data analysis	For descriptive analysis on qualitative information, proportion (%) was used for each item by continents (Africa, Asia, the Americas, Europe and Oceania). For performance indicators on quantitative data, examination coverage, proportion advised further assessment, further assessment participation rate, detection rate, positive predictive value (PPV) of the screening test, and treatment rate were calculated for each programme using the formulas presented in supplementary table S4 (using CanScreen5 website data manager).

For manuscripts utilizing custom algorithms or software that are central to the research but not yet described in published literature, software must be made available to editors and reviewers. We strongly encourage code deposition in a community repository (e.g. GitHub). See the Nature Portfolio guidelines for submitting code & software for further information.

Data

Policy information about availability of data

All manuscripts must include a data availability statement. This statement should provide the following information, where applicable:

- Accession codes, unique identifiers, or web links for publicly available datasets
- A description of any restrictions on data availability
- For clinical datasets or third party data, please ensure that the statement adheres to our policy

The data used in this manuscript are publicly available at the website of CanScreen5 project (https://canscreen5.iarc.fr).

Human research participants

Policy information about studies involving human research participants and Sex and Gender in Research.

Reporting on sex and gender	We did not report data on sex or gender.
Population characteristics	For the quantitative analysis, we indicated the reporting age range for each country.
Recruitment	We collect information and data directly from the Ministry of Health (MoH). IARC's existing network of research collaborators globally is leveraged to reach out to the MoH. The contact person within the MoH is requested to identify the programme coordinators or experts capable of providing reliable information and data. Additionally, the World Health Organization (WHO) regional offices help establish contact with the MoH. In the rare cases in which contact with the MoH cannot be established, academic and/or public health institutes associated with the implementation and evaluation of screening programme are contacted to identify potential data providers.
Ethics oversight	The IARC ethics commettee reviewed the project and waived the requirement for any consent for collecting data. Data providers are mandated to ensure that they have necessary approvals from authorities to share data.

Note that full information on the approval of the study protocol must also be provided in the manuscript.

Field-specific reporting

Please select the one below that is the best fit for your research. If you are not sure, read the appropriate sections before making your selection.

☐ Life sciences ☒ Behavioural & social sciences ☐ Ecological, evolutionary & environmental sciences

For a reference copy of the document with all sections, see nature.com/documents/nr-reporting-summary-flat.pdf

Behavioural & social sciences study design

All studies must disclose on these points even when the disclosure is negative.

Study description	CanScreen5 project was launched in June 2019 and was built upon IARC's successful reporting of the status of implementation and performance of cancer screening programmes in EU Members States. Data collection tools, key performance indicators (KPIs) and strategies for data collection and validation used in the EU project were further adjusted to make these tools globally relevant and suitable for different resource settings. The qualitative data on the programme organization and quantitative data on performance are collected.
Research sample	Ministry of Health or the academic/public health institutes associated with implementation of screening programme are approached to provide data. As of 2022 September, a total of 84 countries joined the project, including 17 countries from Africa, 27 from the Americas, 10 from Asia, 29 from Europe, and 1 (Australia) from Oceania. CanScreen5 is a long-term and dynamic project, and the ideal case is that all countries are willing to join the CanScreen5 project. In the future, more countries will be involved.
Sampling strategy	No sampling strategy was used in this study. We aim to have all countries in the world involved in the CanScreen5 project. Currently, a total of 84 countries joined in the project.
Data collection	The collaborators are given a password-protected access to the data submission platform, from where, the collaborators could provide the qualitative and quantitative data on the cancer screening programme. The researchers were blinded to the study hypothesis.
Timing	June 1 2019-September 1 2022
Data exclusions	7 countries (Antigua and Barbuda, Bulgaria, Dominica, Ecuador, Libya, Saint Kitts and Nevis, and Saint Lucia) were not included in the analysis as they did not fulfil the minimum criteria of having a screening programme for the cancer sites that they have submitted information on.
Non-participation	A total of 60 countries we approached but declined to join the CanScreen5 at this stage. The reasons for non-participation of some countries we approached to participate include; voluntary nature of participation (no national or global mandate), non-availability of approval from higher authorities and reluctance of programmes to share data with the fear of receiving criticisms for poor performance.
Randomization	This is not relevant to the current study. We aim to have more countries to be involved in our project. As long as we have their approval, we will get them involved to provide cancer screening related data.

Reporting for specific materials, systems and methods

We require information from authors about some types of materials, experimental systems and methods used in many studies. Here, indicate whether each material, system or method listed is relevant to your study. If you are not sure if a list item applies to your research, read the appropriate section before selecting a response.

Materials & experimental systems

n/a	Involved in the study
☒	☐ Antibodies
☒	☐ Eukaryotic cell lines
☒	☐ Palaeontology and archaeology
☒	☐ Animals and other organisms
☒	☐ Clinical data
☒	☐ Dual use research of concern

Methods

n/a	Involved in the study
☒	☐ ChIP-seq
☒	☐ Flow cytometry
☒	☐ MRI-based neuroimaging
